# Food insecurity amongst universal credit claimants: the benefits and nutrition study (BEANS), a cross-sectional online study

**DOI:** 10.1007/s00394-025-03596-y

**Published:** 2025-03-10

**Authors:** Michelle Thomas, Peter Rose, Lisa Coneyworth, John Harvey, James Goulding, Juliet Stone, Matt Padley, Patrick O’Reilly, Simon Welham

**Affiliations:** 1 School of Biosciences, Food Nutrition and Dietetics, University of Nottingham, Loughborough, Leicestershire, UK; 2 Centre for Research in Social Policy, Loughborough University, Room U130 Brockington Building, Loughborough, UK; 3 N/LAB, Nottingham University Business School, Jubilee Campus, University of Nottingham, Nottingham, UK; 4 School of Humanities and Social Sciences, John Foster Building, Liverpool John Moores University, Liverpool, UK

**Keywords:** Universal credit, Food insecurity, Diet quality, Minimum income standard

## Abstract

**Purpose:**

Increasing food insecurity (FIS) in the UK presents a major challenge to public health. Universal Credit (UC) claimants are disproportionately impacted by FIS but research on socio-demographic factors and consequent nutritional security is limited.

**Methods:**

A cross-sectional online survey (September 2021 - April 2022) assessed FIS in UC claimants (males and females, *n* = 328) (USDA 10 question module), dietary intake (females, *n* = 43; 3–4 × 24-hour dietary recalls) and coping strategies. Binary logistic regression tested sociodemographic variables influencing the odds of food insecurity. Diets ofUC were compared with national diet and nutrition survey (NDNS) participants and thematic analysis conducted for drivers and impacts of FIS.

**Results:**

FIS was experienced by 84.8% of UC respondents (73.8% very low food security). Equivalised income <£200 week^-1^ increased odds of FIS by 7.3 (3.4–15.3) times compared with households receiving >£300 week^-1^. Being unemployed (*P* = 0.004), travelling > 15 min to obtain food (*P* = 0.016), shopping less than twice per week (*P* = 0.001) and receiving < 47.7% of the minimum income standard (MIS) all increased risk of FIS. Diet quality of working age females was lower (45.9%) compared to those in the NDNS (49.6-55.8%; *P* < 0.05) characterised by limited protein sources, minimal fruit consumption and reliance on bread. Intakes of vitamin A, iron, selenium, potassium, iodine and magnesium were consistently below most NDNS cohorts. Participants felt impotent to make substantive changes to their diets due to poverty.

**Conclusion:**

During this study, dependence on UC almost guaranteed recipients would be food insecure, consuming insufficient micronutrients to support health. MIS may provide a useful benchmark to prevent food poverty.

**Supplementary Information:**

The online version contains supplementary material available at 10.1007/s00394-025-03596-y.

## Introduction

Household food insecurity (FIS) is defined by having limited and uncertain access to adequate food [1] and is currently a significant public health concern. An estimated 7% of UK households (almost 2 million) experienced FIS [2] in the financial year 2021–2022 increasing to 11% in 2022/23 [3]. Food insecurity is typically associated with a range of adverse health conditions including childhood stunting, respiratory disease, obesity and diabetes [4–7], with an estimated cost to the NHS of treating malnutrition of around £19.6 billion annually [8].

Individuals most at risk of food insecurity include lone parents, low-income households, those with low educational attainment, those living with disabilities [9, 10], ill health [11] or caring for someone with a disability [12] and individuals in receipt of benefits [13]. It was estimated that, in the financial year 2021–2022, 31% of households with a claim for universal credit (UC) experienced FIS, equating to 1.27 million households (4.4 times higher than for the general population). However, households in receipt of benefits are often underrepresented in national surveys [14] so the exact level of FIS is difficult to determine.

Universal credit is paid monthly to economically active (working) and inactive households (non-working) to help with living costs. To be able to claim UC people must live in the UK, be aged between 18 and 65 years, although there some exemptions for 16–17 year olds, and have less than £16,000 in savings or investments [15]. The amount of UC a household receives is dependent on the age of the claimant, relationship status, number of children, housing costs and health status (disability and/or health conditions). Working households have their UC reduced by £0.55 for every £1.00 of income earned [16]. In March 2020 at the start of the COVID-19 Pandemic the UK government introduced a £20 a week uplift to UC to help with additional costs encountered during lockdown. This was initially implemented for one year and subsequently extended by a further 6 months before officially ending on the 6th of October 2021. The sudden removal of the uplift is believed to have created instability in households’ economic access to food [17].

There has been relatively limited research on food security and nutrient intake of UC recipients, previous research has focused on the prevalence of food insecurity amongst UC claimants and the factors that influence the likelihood of experiencing food insecurity [18, 19]. Similarly, there is little work assessing how socio-demographic differences amongst UC claimants influence their food security. Since the likelihood of significant damage leading to long-term ill health is particularly high amongst those with infrequent food access, we set out to determine the prevalence and severity of food insecurity among UC claimants and identify factors which might exacerbate and protect against it in this group. We hypothesised that an income from work would be beneficial and that ill health, changes in income (e.g., loss of the uplift in UC), the presence of children and difficulty accessing shops would be detrimental to food security status. Additionally, we aimed to assess the nutrient consumption in UC recipients and compare with the general population. We hypothesised that low incomes would result in a reduced variety of foods and consequent micronutrient composition of their diets.

## Methods

### Study design and participants

An online cross-sectional study was conducted to collect data from adults aged 18–65 years living areas with a high proportion of UC claimants from England, Wales and Scotland who received the £20 week^-1^ uplift. Participants were recruited in two-week blocks at the end of September 2021, February 2022, and April 2022 with targeted advertising (Reach PLC, UK) on Facebook and Instagram. Leaflets were also distributed to local charitable organisations (The Grub Hub, Coventry; Claremont Children and Family Centre, Rugby; The Benn Partnership Centre, Rugby).

Questions from validated surveys [20, 21] collected sociodemographic characteristics, household composition, household income and sources of income, educational achievement, food security status in the previous 30 days (USDA 10 item adult food security module [22]) and coping strategies (e.g. relying on less preferred less expensive foods, borrowing food from a friend or relative and purchasing food on credit or limiting portions size at meal times etc.; Supplementary Fig. 1) [23]. We additionally asked about shopping habits, health and food bank use in the previous seven days (Supplementary Methods). No information about debts, sanctions, repayments or wider support networks was requested. Information was collected using a single online survey hosted by Jisc (Online Surveys tools v2) with a built in consent form and participant information sheet. Food security questions were scored 1 for a positive response and 0 for negative. The food security score was calculated by summing positive responses to the questions. Respondents were “high food secure” (no reported indications of food access problems) if they scored 0, “marginal food secure” (anxiety or worry about having sufficient food in the house, but limited change to the diet) if the score was 1–2, “low food secure” (changes in the quality, variety or desirability of the diet, but food intake not reduced) if the score was 3–5 and “very low food secure” (disrupted eating patterns and reduced food intake) if the score was 6–10 [24]. For analyses, we grouped participants as “food secure” (combined “high” and “marginal”) and “food insecure” (combined “low” and “very low”).

### Minimum income standard assessment

The Minimum Income Standard (MIS) was used in this study as an indicator of the minimum acceptable standard of living to determine how likely benefit recipients were to achieve this standard. The MIS sets out what the public agree everyone in the UK needs for a minimum, socially acceptable standard of living [25]. It is a ‘minimum’ in that it refers to a threshold under which the public agree no one should fall; it is ‘socially acceptable’ in the sense that it is a threshold defined by society [26]. MIS is not just about meeting basic material needs, but critically is about a dignified standard of living that covers all areas of life including social participation and non-material needs. This follows the view set out in the work of Townsend [27], that one critical way in which poverty can be understood, is as a lack of resources relative to what is ordinarily or customarily seen as the approved social norm within any society.

More detailed accounts of the method have been set out previously [28, 29] but in brief, a series of focus groups are undertaken, tasked with establishing minimum needs for each of a range of different household types. Groups comprise members of the public from the household type under discussion, from a range of socio-economic backgrounds, working to a common definition: ‘A minimum standard of living in the UK today includes, but is more than just, food, clothes and shelter. It is about having what you need in order to have the opportunities and choices necessary to participate in society’. This includes defining a realistic, nutritious, socially acceptable diet that allows for participation in customary activities, including additional amounts for seasonal events such as Christmas and birthdays, and modestly eating out occasionally. Focus groups defined menus to mirror these socially acceptable diets, which underwent minor modification by a nutritional expert to ensure that they were nutritionally adequate for that particular group of the population [30]. Minimum budgets are ‘rebased’ every four years for any given household type to ensure that MIS continues to reasonably reflect and capture social norms and expectations.

### Intake24 dietary data

Dietary data was collected using multiple 24-hour dietary recalls using Intake24 between October 2021 - May 2022. Participants who agreed to provide details of their food intake supplied their email address at the end of the online survey. Within 14 days a link to Intake24 for the first food diary was sent via email to participants providing login details and instructions for completion. Upon receipt of the link, participants were asked to complete their food record for the preceding day. Links for subsequent food diary days were sent over the following three to four weeks to either record weekdays (sent Tuesday to Saturday) or weekend days (Sunday or Monday). Only one request for a weekend food diary was sent. The Intake24 procedure involves a computerised 24-hour recall which employs the multiple pass method (intake24.org [31, 32]). Daily energy and nutrient intakes without supplements were calculated and achievement of adequacy (% reference nutrient intake; RNI) determined based on age and sex requirements as per UK dietary recommendations [33]. Foods were matched to the National Diet and Nutrition Survey (NDNS) food sub-categories for use in the analysis.

### NDNS analysis

We used data from the NDNS years 9–11 (rolling programme for year 2016 to 2017 to year 2018 to 2019) for females aged 23–61 years [34]. Datasets were combined for person level dietary data and individual level data and the following variables of interest extracted: sex, equivalised income tertiles, energy and nutrient intakes. The NDNS 9–11 dataset provides tertiles of equivalised income (low, medium and high) rather than specific income amounts or boundaries. Food level dietary data was used for estimating the energy intakes from foods high in fat salt and sugar and used in the calculation of the DQI-I.

### Sensitivity analysis

Misreporting of energy intakes in the BEANs study and NDNS were evaluated using European methodology [35]. The Schofield equation for estimating basal metabolic rate using height and weight was used and Goldberg cut-off points based on Physical Activity level of 1.4 applied. We applied this activity level to the BEANs and NDNS data to provide consistency as activity data was not collected as part of the BEANs study.

### Diet Quality Index-International (DQI-I)

The DQI-I was used to estimate overall diet quality [36]. The four major categories of the DQI-I are: variety (evaluates variety in food groups, and within protein sources), adequacy (assesses foods and nutrients in the diet required in sufficient quantities for a healthy diet), moderation (evaluates food and nutrients in the diet associated with chronic diseases), overall balance (examines the ratio of energy obtained from macronutrients and the ratio of saturated fat to poly and mono-unsaturated fatty acids). The fatty acid ratio was determined by establishing the total consumption of polyunsaturated fatty acids (PUFA), monounsaturated fatty acids (MUFA) and saturated fatty acids (SFA), establishing their relative abundance and presenting as a ratio (PUFA: MUFA: SFA). The maximum DQI-I score is 100 being derived from the sum of sub-category criteria in each main category. Variety accounts for 20%, adequacy 40%, moderation 30% and overall balance 10%.

The adequacy category concerns dietary components required in sufficient quantities for a healthy diet. The recommended quantities of vegetables, fruit, grains, and fibre is dependent on energy intake (kcal) [36]. In this study we used the energy recommendations of the DQI-I and created the groups < 1700 kcal, >=1700 - <2700 kcal and > = 2700 kcal for scoring. (Supplementary Table 1). Weights applied to servings in food groups can be found in Supplementary Table 2. We used the Eatwell guide limitations for foods high in fat, salt and sugar [37].

### Qualitative Data regarding perceptions of impact of income changes on diet

Information concerning perceptions of participants towards the impact of changes in income on their food consumption patterns was gathered using an open-ended question which asked them to state in their own words how they felt that their income impacted on their diet. This was to aid understanding of how responses to changes in income are reflected in nutritional outcomes [38]. The question was necessarily left open to facilitate participant ability to express their experiences in their own words and avoid the predefinition of acceptable answers.

### Statistical analyses

All analysis was completed in SPSS version 29 IBM Corp. Released 2023. IBM SPSS Statistics for Windows, Version 29.0.2.0 Armonk, NY: IBM Corp. Statistical significance was at the *P* < 0.05 level. Descriptive analysis of sociodemographic characteristics and food security status were conducted using chi sq and are presented as frequency and percentages for the total population across the food security domains and by dichotomised food security status (food secure vs. food insecure). Binary logistic regression was used for predicting the probability of food insecurity and increasing severity of food insecurity. The predictor variables were categorical using the group with fewest food insecure as the reference group. Normality of dietary data was assessed using Shapiro-Wilk test. Parametric variables are presented as means and S.E.M. non-parametric variables are presented as medians (25th and 75th percentile).We present the frequency and percentages of participants within each of the sub-categories of the DQI-I and estimates of the total score achieved in each of the sub-categories of the DQI-I using mean ± S.E.M. However, non-parametric tests were performed because the scoring system is ordinal and not continuous. Micronutrient data are presented as the frequency and percentage of females below the Lower Reference Nutrient Intake (LRNI) and the percentage of the Reference Nutrient intake (RNI).

### Analysis of qualitative responses

A multistage analysis of the responses to our open ended question on perceptions was undertaken to identify the prevalence of key themes. This involved a preliminary statistical analysis using “wordstats” text analysis software (Provalis Research, Montreal, Canada) to perform multivariate analysis of the content of responses. This performed basic word and term frequency counts with subsequent clustering of word occurrences to generate observations as to the overall shapes and core concerns of respondents. This helped to guide an interpretive thematic analysis of all 296 responses which focused individual themes into two domains; impact and responses.

## Results

### Population demographics and experience of food insecurity

A total of 328 participants met the inclusion criteria and provided complete responses. Respondents comprised households with two adults only (∼ 8%), one adult only (∼ 41%), two adults with children (∼ 20%) and one adult with children (31%; Table 1). Average age was 41.5 ± 10,7 years, most were female (> 70%), British (> 90%) and either single or divorced, separated, or widowed (> 70%; Table 1). The level of education was reasonably high with > 30% having achieved level 4 and above, 73% were not in paid employment. Most (> 60%) were overweight with two fifths being obese, while ∼ 6% were underweight (Supplementary Table 3). The prevalence of food insecurity was far greater than we had anticipated. Almost all (84.4%) were food insecure, with most (73.8%) experiencing “very low food security”.

**Table 1:** Participants socio-demographic characteristics across the food security domains and dichotomized by food security status (food secure and food insecure)

	Total		High		Marginal		Low		Very low		*P* value	Food secure		Food insecure		*P* value
	n	%	n	%	n	%	n	%	n	%		n	%	n	%	
**Total population**	328	100	25	7.6	25	7.6	36	11.0	242	73.8		50	15.2	278	84.8	
**Gender**											0.275^1^					0.252^1^
Male	86	26.2	6	7.0	11	12.8	9	11.0	60	69.8		17	19.8	69	80.2	
Female	239	72.9	18	75	14	5.9	27	11.3	180	75.3		32	13.4	207	86.6	
Other	3	0.9	1	33.3	0	0.00	0	0.00	2	66.7		1	33.3	2	66.7	
**Age groups (years)**											0.399					0.603
19–35	101	30.9	5	5.0	11	10.9	11	10.9	74	73.3		16	15.8	85	84.2	
36–49	147	45.0	12	8.2	7	4.8	14	9.5	114	77.6		19	12.9	128	87.1	
50+	79	24.2	8	10.1	6	7.6	11	13.9	54	68.4		14	17.7	65	82.3	
**Total**	**327**															
**Education**											0.318^1^					0.238^1^
level 7/8	20	6.1	0	0.0	2	10.0	4	20.0	14	70.0		2	10.00	18	90.0	
level 4–6	92	28.0	13	14.1	9	9.8	8	8.7	62	67.4		22	23.91	70	76.1	
level 3	66	20.1	1	1.5	6	9.1	7	10.6	52	78.8		7	10.61	59	89.4	
level 2	62	18.9	7	11.3	3	4.8	8	12.9	44	71.0		10	16.13	52	83.9	
level 1	13	4.0	2	15.4	0	0.0	1	7.7	10	76.9		2	15.38	11	84.6	
Other qualification	16	4.9	1	6.3	1	6.3	1	6.3	13	81.3		2	12.50	14	87.5	
None	51	15.5	1	2.0	3	5.9	5	9.8	42	82.4		4	7.84	47	92.2	
Prefer not to say	8	2.4	0	0.0	1	12.5	2	25.0	5	62.5		1	12.5	7	87.5	
**Economical active**											**0.030**					**0.006**
yes	89	27.1	11	12.4	11	12.4	7	7.9	60	67.4		22	24.7	67	75.3	
no	239	72.9	14	5.9	14	5.9	29	12.1	182	76.2		28	11.7	211	88.3	
**Income source**											**0.01**					**0.001** ^**2**^
UC only	222	67.7	13	5.9	11	5.0	27	12.2	171	77.0		24	10.8	198	89.2	
Employment + UC	106	32.3	12	11.3	14	13.2	9	8.5	71	67.0		26	24.5	80	75.5	
**EQVINC**											**< 0.001** ^1^					**< 0.001**
<£200 per week	198	60.4	7	3.5	6	3.0	17	8.6	168	84.8		13	6.6	185	93.4	
£200 -£300 per week	59	18.0	5	8.5	8	13.6	6	10.2	40	67.8		13	22.0	46	78.0	
>£300 per week	71	21.6	13	18.3	11	15.5	13	18.3	34	47.9		24	33.8	47	66.2	
**EQVFS**											0.292^1^					0.063
£0-£30 per week	98	29.9	5	5.1	4	4.1	12	12.2	77	78.6		9	9.2	89	90.8	
£30-£60 per week	87	26.5	7	8.0	8	9.2	6	6.9	66	75.9		15	17.2	72	82.8	
£60-£90 per week	92	28.0	6	6.5	7	7.6	14	15.2	65	70.7		13	14.1	79	85.9	
>£90 per week	51	15.5	7	13.7	6	11.8	4	7.8	34	66.7		13	25.5	38	74.5	
**Percentage of income spent on food**											**0.028**					**0.004**
< 15%	57	17.4	8	14.0	9	15.8	8	14.0	32	56.1		17	29.8	40	70.2	
15–30%	127	38.7	6	4.7	8	6.3	17	13.4	96	75.6		14	11.0	113	89.0	
30–60%	68	20.7	6	8.8	6	8.8	4	5.9	52	76.5		12	17.6	56	82.4	
> 60%	76	23.2	5	6.6	2	2.6	7	9.2	62	81.6		7	9.2	69	90.8	
**Ethnicity**											**0.048** ^1^					0.312^12^
White	306	93.3	24	7.8	21	6.9	31	10.1	230	75.2		45	14.7	261	85.3	
Not white	22	6.7	1	4.5	4	18.2	5	22.7	12	54.5		5	22.7	17	77.3	
**Relationship status**											**0.006**					0.568^2^
Couple	93	28.4	1	1.1	11	11.8	7	7.5	74	79.6		12	12.9	81	87.1	
Single	235	71.6	24	10.2	14	6.0	29	12.3	168	71.5		38	16.2	197	83.8	
**Household Composition**											**0.065** ^**1**^					0.344^1^
2 adult no children	26	7.9	0	0.0	3	11.5	2	7.7	21	80.8		3	11.5	23	88.5	
1 adult no children	133	40.5	10	7.5	7	5.3	16	12.0	100	75.2		17	12.8	116	87.2	
2 adult with children	67	20.4	1	1.5	8	11.9	5	7.5	53	79.1	**0.019**	9	13.4	58	86.6	
1 adult with children	102	31.1	14	13.7	7	6.9	13	12.7	68	66.7		21	20.6	81	79.4	
**Accommodation**											**< 0.001** ^**1**^					0.197^1^
Own outright	10	3.0	3	30.0	0	0.0	1	10.0	6	60.0		3	30.0	7	70.0	
Mortgage/shared ownership	23	7.0	2	8.7	1	4.3	2	8.7	18	78.3		3	13.0	20	87.0	
Rent	277	84.5	16	5.8	24	8.7	27	9.7	210	75.8		40	14.4	237	85.6	
Live here rent free	12	3.7	4	33.3	0	0.0	3	25.0	5	41.7		4	33.3	8	66.7	
Other	6	1.8	0	0.0	0	0.0	3	50.0	3	50.0		0	0.0	6	100.0	

1 = cells have expected count less than 5

2 = P value for a 2 × 2 table, Continuity Correction

Chi square test of independence

Education levels include:

Level 1 GCSE- grades 3, 2, 1 or grades D, E,F, G, level 1 National Vocational Qualification (NVQ); Level 2, GCSE- Grades 9, 8, 7, 6, 5, 4 or grades a*, A, B, C, NVQ level 2; Level 3, A Level, As Level, NVQ level 3; Level 4, Certificate of Higher Education (CertHE), Higher National Certificate (HNC), Level 4 NVQ; Level 5, Diploma of higher education (DipHE), foundation degree, Level 5 NVQ; Level 6, Degree level, Level 6 NVQ; Level 7, Master’s degree, Post graduate certificate in education (PGCE), Level 7 NVQ; Level 8, Doctorate

EQVINC - Equivalised household income. Equivalisation is a standard method to give each household member a representative income. It is calculated by dividing household income by the household composition weighting derived from the McClements score

EQVFS- Equivalised food spend is the household food spend which has equivalised by dividing by the score for household composition

**Fig. 1:**
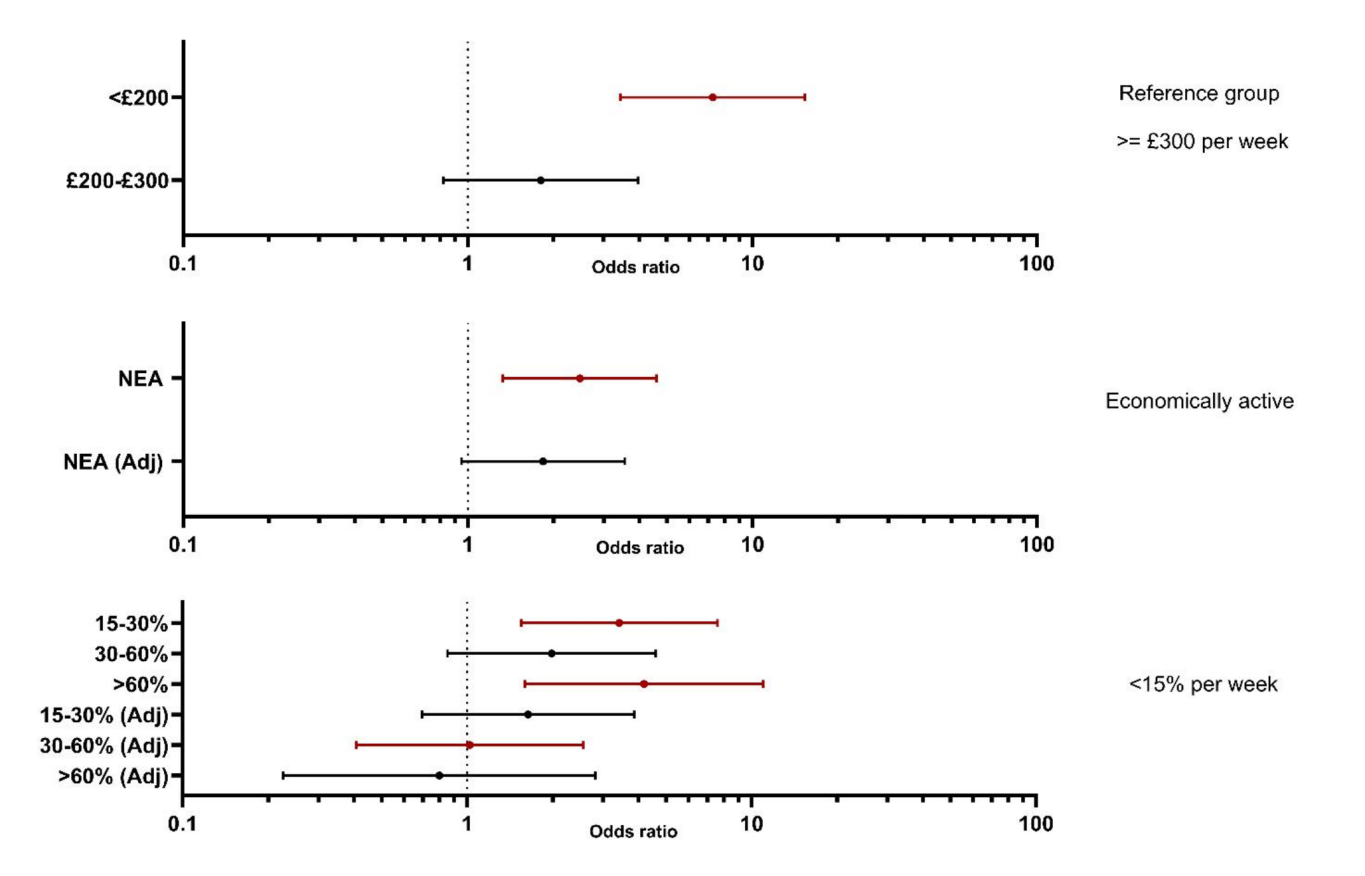
Binary logistic regression predicating odds of FIS amongst Universal Credit claimants compared to food secure Universal Credit claimants. Top figure shows the odds ratio of FIS based on household income per week (reference group of £300 per week), middle figure shows odds of FIS dependent on economic activity (Reference group economically active) and bottom figure depicts the percentage of equivalized income required for equivalized food spend per week Reference group < 15% per week) Crude and equivalized income adjusted Odds ratios (95% CI) presented. NEA = Non-Economical Active Adj = Adjusted

**Fig. 2:**
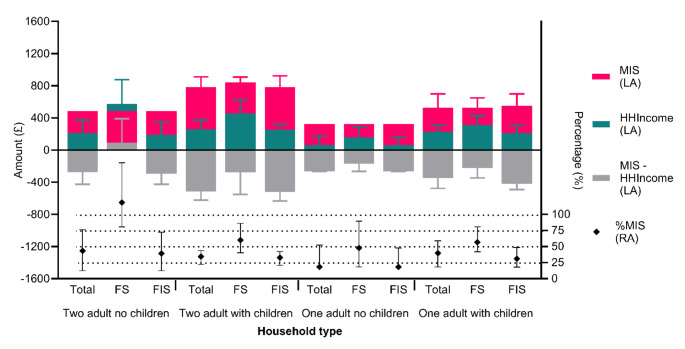
Weekly household income (blue bar) by household type and food security status compared to the UK public recommendations for adequate household income (Minimum Income Standards (pink bar)). Food insecure households across all household’s type met a lower percentage of the minimum income standards (black symbol). Median 25th and 75th Percentile shown, MIS = Minimum Income Standard, RA = Right Axis, LA = Left Axis, FS = Food Secure, FIS = Food Insecure

Household equivalised income was just £150.72 week^− 1^ (£98.36 - £282.15; Median UK equivalised household income in 2021 was £603 week^− 1^ [39]). Food insecurity was most likely for those with an equivalised household income below £200 week^− 1^ (*P* < 0.001), without work (*P* = 0.006) and who spent > 15% of household income on food (*P* = 0.004). Once income dropped below the £200 threshold, the odds of experiencing food insecurity leapt **7 fold** (*P* = 0.001; Fig. 1) in comparison with those on higher incomes, and the likelihood of being severely food insecure by **> 6 times**. Household incomes represented < 50% of that required to meet the MIS for all food insecure participants (Fig. 2). ROC analysis indicated that food insecurity was best predicted at 47.681% of MIS (AUC = 0.729), being 5.563 (2.903–10.658; *p* < 0.001) times more likely if respondents received less than this threshold compared with those with an income above it. However, whilst few people exceeded this income (∼ 20%) at no point did the proportion experiencing food insecurity drop below 50% and this level of FS was only achieved for those exceeding 80% of the MIS.

Being economically inactive increased the odds of experiencing food insecurity (OR 2.474 [1.328–4.611]; *P* = 0.004) but the benefit of employment was negated if incomes were not sufficiently high (*P* = 0.071; Fig. 1). Spending above 15% of household income on food was associated with significantly increased odds of food insecurity, but again, this was income dependent, showing reduced odds with increasing income. The presence of children in the household did not greatly influence the prevalence of food insecurity, however the number of adults did. Remarkably, while 24 (7.3%) single adults (with or without children) were classified as having high food security, this was only the case for 1 respondent (0.3%) from a 2-adult household.

**Fig. 3:**
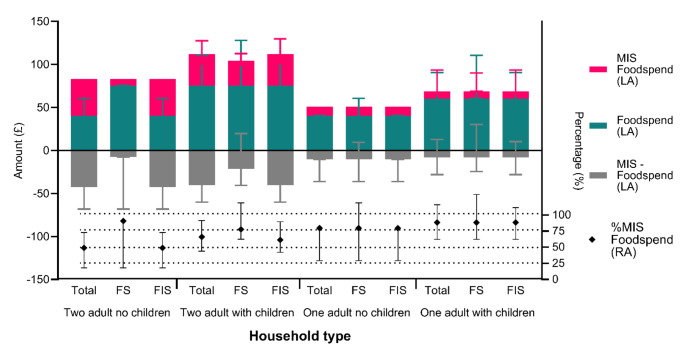
Weekly food spend (blue bar) by household type and food security status compared to the UK public recommendations for adequate food spend (derived from the Minimum Income Standards (pink bar)). Food insecure two adult households with and without children met a lower percentage of the minimum income standards recommendations (black symbol). Median 25th and 75th Percentile shown, MIS = Minimum Income Standard, RA = Right Axis, LA = Left Axis, FS = Food Secure, FIS = Food Insecure

The overall proportion of income spent on food was 1.8 times the national average (14.4% [40]) at 26.1% (19.5-52.3%), rising to 2.3 times for those with children (33.1% [19.5–67.5%]). It was notable that just over a quarter of food insecure households had to spend more than half of their household income on food. Comparison of food spending indicated that households comprising a single adult with children came closest to achieving the level of expenditure required to meet the minimum living standards (96% of expenditure level; [41]; Fig. 3). For others, this ranged from 61 − 76%, with two adult households being least able to achieve necessary expenditure levels. Distance of travel to obtain food additionally impacted food insecurity with those required to travel > 15 min being 2.157 (1.155–4.027) times more likely to be FIS than those with shorter journey times (*P* = 0.016).

### Frequency of adoption of extreme coping mechanisms

A range of coping strategies were adopted by those experiencing food insecurity (Supplementary Fig. 1). Those most commonly employed included relying on less preferred and less expensive foods (92.1%), reducing the quantity (89.6%) and variety (87.8%) of fruit and vegetable intake, borrowing food or relying on help from friends or relatives (63.3%), restricting portion sizes at mealtimes (81.7%) and reducing food variation in meals (84.2%). For those with children, 75.5% reported restricting consumption by adults in order to enable small children to eat. Strikingly, over half of the very low food insecure group went entire days without eating.

In addition to the significant impact of dependence on UC, removal of the £20 uplift coincided with an increase in extreme coping and a decrease in dietary quality/diversity. Most respondents (58.5%) felt that the uplift had enabled an increase in fruit and vegetable consumption, but that this was reduced with its loss (66.9% of participants) and had negatively impacted their ability to purchase meat (64.6%), seafood (53.0%) and even dairy products (41.5%;).

### Diet quality comparisons between UC and non-UC cohorts

Diet quality was assessed in females (few males recorded intakes, so were excluded) and compared outcomes with those from the general population using NDNS data grouped by household income tertile (EQV1-EQV3; lowest to highest). Overall DQI-I scores were lower for BEANS participants compared with all NDNS EQVs (4.5-13.6% lower; Table 2) due principally to limited variety (up to 18.8% less; *P* < 0.001) and adequacy (5.1-19.8% less; *P* < 0.001). Nearly 60% of the NDNS cohort recorded consuming 2 or more servings of vegetables. This was only achieved by 18.6% of BEANS participants who additionally consumed few if any fruit (79.0% consumed 1 or none; Table 2). 16% of BEANS participants did not record a single serving of vegetables. This was the case for just 3.5% of EQV1 and one% or less of the EQV2 or EQV3 respondents.

**Table 2:** Comparison of Diet Quality Index-International (DQI-I) and percentage within subcategories for females aged 23–61 years with an income from Universal Credit (BEANs) and per the criteria for equivalised income tertiles in the National Diet and Nutrition Survey (years 9–11)

Component	Score	Points	Scoring Criteria	BEANs		EQV1			EQV 2			EQV 3^4^		
				(n = 43)		(n = 202)			(n = 202)			(n = 249)		
Overall DQI (Diet Quality Index) Score ^Ŧ^	0-100			Mean	S.E.M	Mean	S.E.M	p value	Mean	S.E.M	p value	Mean	S.E.M	p value
				49.3	1.37	51.6^a^	0.71	0.164	54.4^b^	0.66	0.001	57.1^c^	0.54	<.001
Variety	0–20			n	%	n	%		n n	%		n n	%	
Overall food group variety ^¥^	0–15	15	≥ 1 serving from each food groups	2	4.7	26	12.9	0.273	45	22.3	0.003	81	32.5	<.001
		12	Any 1 food missing/d	14	32.6	77	38.1		80	39.6		99	39.8	
		9	Any 2 food groups missing/d	18	41.9	68	33.7		56	27.7		51	20.5	
		6	Any 3 food groups missing/d	5	11.6	23	11.4		18	8.9		13	5.2	
		3	≥ 4 food groups missing/d	4	9.3	8.	4.0		3	1.5		5.	2.0	
		0	None from any food groups	2	4.7	26	12.9		45	22.3		81	32.5	
Within-group variety form protein source ^¥^	0–5	5	≥ 3 different sources/d	12	27.9	60	29.7	0.020	61	30.2	0.001	67	26.9	0.069
		3	2 different sources/d	15	34.9	86	42.6		94	46.5		123	49.4	
		1	From 1 source/d	13	30.2	55	27.2		47	23.3		55	22.1	
		0	None	3	7.0	1	0.5		0	0.0		4	1.6	
**Adequacy**	**0–40**													
Vegetable group ^1¥^	0–5		**≥ 3–5 servings/d = 5, 0 servings = 0**											
		5	≥ 100%	1	2.3	20	9.9	<.001	30	14.9	<.001	62	24.9	<.001
		3	< 100 − 50%	7	16.3	62	30.7		87	43.1		108	43.4	
		1	< 50%	28	65.1	113	55.9		83	41.1		78	31.3	
		0	0	7	16.3	7	3.5		2	1		1	0.4	
Fruit group ^1¥^	0–5		**≥ 2–4 servings/d = 5, 0 servings = 0**											
		5	≥ 100%	3	7	32	15.8	0.479	39	19.3	0.013	74	29.7	<.001
		3	< 100 − 50%	6	14	28	13.9		33	16.3		60	24.1	
		1	< 50%	17	39.5	76	37.6		93	46		96	38.6	
		0	0	17	39.5	66	32.7		37	18.3		19	7.6	
Grain Group ^1¥^	0–5		**≥ 6–11 servings/d = 5, 0 servings/d = 0**											
		5	≥ 100%	4	9.3	0	0	<.001	3	1.5	<.001	2	0.8	<.001
		3	< 100 − 50%	16	37.2	47	23.3		38	18.8		40	16.1	
		1	< 50%	23	53.5	152	75.2		159	78.7		203	81.5	
		0	0	0	0	3	1.5		2	1		4	1.6	
Fibre ^1¥^	0–5		**≥ 20-39g/d = 5, 0 g/d = 0**											
		5	≥ 100% = 5	8	18.6	43	21.3	0.271	56	27.7	0.04	112	45.0	<.001
		3	< 100 − 50%	24	55.8	128	63.4		123	60.9		130	52.2	
		1	< 50%	11	25.6	31	15.3		23	11.4		7	2.8	
		0	0	0	0	0	0		0	0		0	0	
Protein^¥^	0–5		**≥ 10% of energy/d = 5, 0% of energy/d = 0**											
		5	≥ 100%	42	97.7	199	98.5	0.693	200	99.0	0.047	247	99.2	0.361
		3	< 100 − 50%	1	2.3	3	1.5		2	1.0		2	0.8	
		1	< 50%	0	0	0	0		0	0		0	0	
		0	0	0	0	0	0		0	0		0	0	
Iron ^2¥^	0–5		**100% RNI = 5, 0% of energy/d = 0**											
		5	≥ 100%	4	9.3	21	10.4	0.296	31	15.3	0.005	68	27.3	<.001
		3	< 100 − 50%	21	48.8	121	59.9		133	65.8		143	57.4	
		1	< 50%	18	41.9	60	29.7		38	18.8		38	15.3	
Calcium^¥^	0–5		**100% RNI = 5, 0% of energy/d = 0**											
		5	≥ 100%	24	55.8	86	42.6	0.144	96	47.5	0.120	150	60.2	0.006
		3	< 100 − 50%	14	32.6	99	49.0		95	47.0		94	37.8	
		1	< 50%	5	11.6	17	8.4		11	5.4		5	2.0	
Vitamin C^¥^	0–5		**100% RNI = 5, 0% of energy/d = 0**											
		5	≥ 100%	22	51.2	127	62.9	0.263	158	78.2	0.001	222	89.2	<.001
		3	< 100 − 50%	14	32.6	56	27.7		32	15.8		25	10.0	
		1	< 50%	7	16.3	19	9.4		12	5.9		2	0.8	
**Moderation**	**0–30**													
Total fat ^¥^	0–6	6	≤ 20% of total energy/d	3	7	6	3.0	0.402	4	2.0	0.195	4	1.6	0.096
		3	> 20–30% of total energy/d	11	25.6	47	23.3		50	24.8		60	24.1	
		0	> 30% of total energy/d	29	67.4	149	73.8		148	73.3		185	74.3	
Saturated fat^¥^	0–6	6	≤ 7% of total energy/d	3	7	15	7.4	0.985	9	4.5	0.451	12	4.8	0.807
		3	> 7–10% of total energy/d	7	16.3	31	15.3		50	24.8		46	18.5	
		0	> 10% of total energy	33	76.7	156	77.2		143	70.8		191	76.7	
Cholesterol ^3¥^	0–6	6	≤ 300 mg/d	40	93.0	188	93.1	0.997	188	93.1	0.997	231	92.8	0.998
		3	> 300–400 mg/d	2	4.7	9	4.5		9	4.5		12	4.8	
		0	> 400 mg/d	1	2.3	5	2.5		5	2.5		6	2.4	
Sodium^¥^	0–6	6	≤ 2400 mg/d	37	86	179	88.6	0.589	172	85.1	0.495	204	81.9	0.324
		3	> 2400–3400 mg/d	4	9.3	19	9.4		26	12.9		40	16.1	
		0	> 3400 mg/d	2	4.7	4	2.0		4	2.0		5	2.0	
Empty calorie Foods ^¥^	0–6	6	≤ 3% of total energy/d	3	7	16	7.9	0.967	18	8.9	0.914	24	9.6	0.786
		3	> 3–10% of total energy/d	11	25.6	49	24.3		52	25.7		55	22.1	
		0	> 10% of total energy/d	29	67.4	137	67.8		132	65.3		170	68.3	
**Overall Balance** ^5^	**0–10**													
Macronutrient ratio	0–6	6	[55–65]: [10–15]: [15–25]	0	0	1	0.5	< 0.643	0	0.0	0.028	0	0.0	0.006
(Carbohydrate: protein: fat from energy) ¥		4	[52–68]: [9–16]: [13–27]	3	7	7	3.5		2	1		2	0.8	
		2	[50–70]: [8–17]: [12–30]	4	9.3	14	6.9		12	5.9		11	4.4	
		0	otherwise	36	83.7	180	89.1		188	93.1		236	94.8	
Fatty acid ratio (PUFA: MUFA: SFA)^4 ¥^	0–4	4	P/S = [1-1.5] and M/S = [ 1-1.5]	0	0	4	2.0	0.360	3	1.5	0.238	4	1.6	0.277
		2	P/S = [0.8–1.7] and M/S = [0.8–1.7]	1	2.3	13	6.4		18	8.9		20	8.0	
		0	Otherwise = 0	42	97.7	185	91.6		181	89.6		225	90.4	

Table adapted from Kim, Soowon et al. [36]

1, Based on calorie intake groups < 1700 kcal, >=1700-<2700, >=2700

2, Based on the RNI age specific

3, No value for Cholesterol with the NDNS data set- an average of the BEANs results applied to the NDNS participants

4, Abbreviations PUFA- polyunsaturated fatty acids. MUFA- monounsaturated fatty acids. SFA- saturated fatty acids; P/S ratio of PUFA to SFA. M/S ratio of MUFA to SFA

EQV- equivalised income tertiles. EQV1 lowest, EQV2 middle, EQV3 highest

5, Ratio of percentage energy from carbohydrate to protein to fat

Ŧ, independent T Test used to test for differences between BEANs and Lowest, Middle, and Highest NDNS tertiles

¥, Chi Square test used to test for difference in the frequency of participants within the subcomponents categories compared to BEANs

BEANS participants’ diets included the fewest food groups, with only 62.8% consuming 2 or more different sources of protein, whilst for those in EQV1 and EQV2, approximately 70% had 2 or more sources, increasing to 76.3% for EQV3 (*P* = 0.001). Intake of almost all macronutrients and micronutrients was lower than for NDNS cohorts and, contrary to expectations, this was true even for those more commonly increased in low income groups including fat and saturated fat (Supplementary Table 4). Recording of very low (“non-plausible”) energy intakes was very frequent among BEANS participants (46%) and considerably more so than for NDNS respondents (Supplementary Table 5). Similarly, protein intake was low compared with NDNS tertiles. The only dietary component which showed elevated intakes for BEANS compared to NDNS groups were cereal products which showed a higher grain consumption (*P* < 0.001 cf. all groups). However, this was solely due to elevated consumption of white bread (52.2 g day^− 1^ [17.8–92.4]) which was around 1.5–2.5 times higher than for all NDNS income tertiles (21.3–33.6 g day^− 1^; *P* < 0.001).

Micronutrient intakes of BEANS participants were lower than those of EQV3 for all except calcium and sodium (P *≤* 0.04; Supplementary Table 6). Comparisons with EQV2 showed significantly reduced intakes of vitamins A, B6, C and B12, thiamine, niacin, folate, potassium, magnesium, copper, zinc, iodine, selenium and iron (Table 3). While levels for EQV1 were more similar to BEANS, their intakes remained higher and for vitamin A, selenium and iron, this was significantly so. Micronutrient intakes for EQV1 were consistently lower than those of the higher NDNS income brackets (*P* < 0.001). The number of BEANS respondents consuming less than the lower reference nutrient intake (LRNI; sufficient for only 2.5% of the population) were consistently greater than for NDNS cohorts (Table 3).

**Table 3:** Comparison of micronutrient intakes females aged 23–61 years between BEANS participants and NDNS income tertiles. Percentage of the reference nutrient intake (RNI) achieved and proportion of the population with intakes below the Lower reference nutrients intake (LRNI)

	Beans (*n* = 43)		EQV 1(*n* = 202)				EQV 2 (*n* = 202)				EQV 3 (*n* = 249)			
**Nutrient**	% < LRNI	Median % RNI	% < LRNI	Median % RNI	P value^1^ LRNI	P value ^2^ % RNI	% < LRNI	Median % RNI	P value^1^ LRNI	P value ^2^% RNI	% < LRNI	Median % RNI	P value^1^ LRNI	P value ^2^ % RNI
Riboflavin	25.6	112.7	15.3	110.9	0.106	0.455	12.9	118.3	**0.035**	0.136	7.2	135.4	**< 0.001**	**< 0.001**
Vitamin A	16.3	68.5	12.9	94.1	0.552	**0.006**	8.9	109.4	0.147	**< 0.001**	3.6	135.3	**< 0.001**	**< 0.001**
Folate	11.6	84.6	11.9	86.2	0.963	0.117	6.4	94.7	0.236	**0.003**	2.8	109.6	**0.07**	**< 0.001**
Vitamin B12	4.7	233.6	2.5	250	0.437	0.147	0.5	263	**0.024**	**0.021**	2.0	299.4	0.295	**0.002**
Vitamin C	7.0	108.7	1.5	125	**0.034**	0.456	1.0	159.9	**0.012**	**0.015**	0.0	202.4	**0.001**	**< 0.001**
Vitamin B 6	n/a	106.0	0.0	110	n/a	0.146	0.5	118.3	n/a	**0.012**	0.0	127.8	n/a	**< 0.001**
Thiamine	0.0	142.3	0.0	151	n/a	0.502	0.0	161.8	n/a	**0.029**	0.0	176.1	n/a	**< 0.001**
Selenium	69.8	46.3	55.9	63.1	0.095	**0.008**	49.5	66.8	**0.016**	**< 0.001**	38.2	75.8	**< 0.001**	**< 0.001**
Iron < = 50 yrs.^$^	52.8	53.1	42.4	57.4	0.353	**0.041**	31.9	62.4	**0.042**	**0.003**	26.3	68.7	0.006	**< 0.001**
Iron > 50 yrs.^$$^	28.6	72.0	18.2	84.6	0.520	0.546	8.2	99.6	0.093	0.074	1.4	112.7	**0.047**	**< 0.001**
Potassium	37.2	62.8	31.7	65	0.483	0.574	23.3	70.9	0.058	**0.017**	14.1	78.8	**< 0.001**	**< 0.001**
Iodine	32.6	74.4	17.3	79.9	**0.023**	0.277	9.4	88.9	**< 0.001**	**0.026**	4.8	100.3	**< 0.001**	**< 0.001**
Magnesium	23.3	75.5	16.8	75.9	0.319	0.829	10.4	85.0	**0.021**	**0.037**	4.4	96.5	≤ **0.001**	**< 0.001**
Calcium	16.3	102.1	12.4	92.6	0.490	0.677	8.4	97.0	0.115	0.996	0.0	110.5	**0.008**	**0.009**
Zinc	20.9	91.8	8.4	97.6	**0.045**	0.257	6.4	105.2	**0.010**	**0.030**	n/a	114.1	**< 0.001**	**< 0.001**
Sodium	0.0	103.2	n/a	n/a	n/a	n/a	n/a	n/a	n/a	n/a	n/a	n/a	n/a	n/a
Copper	n/a	70.9	n/a	73.3	n/a	0.294	n/a	83	n/a	0.010	n/a	95.3	n/a	**< 0.001**

1Chi sq test to understand difference in representative of population groups with and intake below the lower reference nutrient intake

2 Mann Whitney test to determine differences in % Reference Nutrient Intake achieved (RNI)

$ Number of participants in study under 50 years BEANS (*n* = 36), NDNS Tertiles 1 (*n* = 158), NDNS tertiles 2 (*n* = 158), NDNS tertiles 3 (*n* = 175)

$$ Number of participants in study over 50 years BEANS (*n* = 7), NDNS Tertiles 1 (*n* = 44), NDNS tertiles 2 (*n* = 44), NDNS tertiles 3 (*n* = 74)

EQV 1, equivalised income tertile 1 (lowest), EQV 2, equivalised income tertile 2 (middle), EQV, equivalised income tertile 3 (highest)

Because of the increase in bread consumption, we examined the relative importance of cereals for mineral provision. Cereal products were significant contributors to mineral intakes among BEANS participants, providing 28.0% of zinc (1.6 mg d^− 1^ [1.0 mg d^− 1^- 2.9 mg d^− 1^]), 49.8% of iron (3.7 mg d^− 1^ [2.3–5.2 mg d^− 1^]) and 33.0% of dietary selenium (9.9 µg d^− 1^ [6.1 µg d^− 1^– 14.0 µg d^− 1^]). Milk was also a significant source of zinc in the diet of BEANs participants supplying 11.5% (0.7 mg d^− 1^ [0.4 mg d^− 1^- 1.0 mg d^− 1^]), being almost twice the proportion, it contributed in NDNS cohorts (6.2% [2.2–10.8%]). Meat products remained relatively important sources of zinc (16.5% with processed meat products being the highest contributors) and selenium intakes (15%). Meat represented a less important contributor of iron, accounting for just 7.4% (0.5 mg d^− 1^ [0.1 mg d^− 1^ -1.6 mg d^− 1^).

### Awareness of negative health impacts of necessary dietary choices

A higher prevalence of FIS was observed among respondents who rated their health as fair or bad (Supplementary Table 3). This was reflected in qualitative responses which indicated that awareness of the negative health impacts of dietary “choices” was widespread and provoked considerable concern.

#### Domain 1, Impact of income on diet

In the initial textual analysis, a cluster of words and terms associated with low income and food quality was the most prevalent (26% of responses). Other prominent clusters related to cheaper products, reduction in meat and fruit consumption and resorting to less healthy ready meals. Thematic analysis indicated a widespread belief that loss of the uplift had negatively impacted nutritional wellbeing, with very few stating no impact. The vast majority of respondents indicated that the loss of the extra payment affected what they could afford to buy in ways which adversely impacted their levels of choice and variety of food. Reductions in the consumption of meats, fruit and vegetables were frequently identified.

#### Domain 2. Responses to the impact of income on diet

Many responses voiced specific concerns about the adverse health consequences of these decisions with a smaller number of people mentioning links to existing conditions or vulnerabilities.

Among the key concerns in this respect was an inability to buy correct food or eat frequently enough, thereby exacerbating health issues;

Both myself and my husband are diabetic and trying to have a healthy diet on a low income is very difficult as we can’t afford the more expensive healthier options.

I have food aversions and dietary restrictions and this leaves me with not much food I can eat that is healthy for me. I am lucky enough to have enough to survive, but it’s taking its toll.

Further health issues were linked to resorting to what some described as a “beige” diet, with more carbohydrates consumed in order to provide people with filling food, supporting observations of elevated bread consumption. Regarding the practice of skipping meals, it was generally recognised that this was a bad option but this view was closely linked with the feeling that they had little choice.

## Discussion

In this study we show that, between October 2021 and April 2022, food insecurity among UC recipients was rife with almost 70% experiencing “very low food security”, a proportion far in excess of previously recorded values. Respondents were frequently forced to adopt extreme coping strategies including reducing meal size and frequency along with starving for entire days (43.6% of the cohort). Diet diversity was extremely poor, driven by consumption of a limited variety of protein sources, low frequency of fruit and vegetable consumption and a reliance on starchy carbohydrates. In addition the proportion of females with micronutrient intakes below the LRNI was higher than was seen for the general population. On the whole, people recognised that their diets and coping strategies were undesirable and linked to poor nutrition and a decline in health, but felt impotent to make any positive change. The overriding cause of the challenges described was poverty.

The level of food insecurity that we observed was substantially higher than those recorded in recent national datasets [42, 43]. The Family Resource Survey (FRS) released in March 2021 [42] for the financial year 2020–2021, indicated that 27% of people with an income from UC were food insecure and of those, 15% had “very low food security” [42]. Targeting of FRS participants was by random postcode selection, with those choosing to participate completing an interviewer led questionnaire. While helpful for enabling acquisition of detail, this approach can reduce respondent anonymity and is known to reduce both uptake and likelihood of complete responses to questions considered shameful [44–46]. Hence, there is potential that lower estimates of FIS and reporting of severe coping strategies would be diminished. In the current study, responses were anonymous, engaged through advertisements and not interviewer led. The Food Foundation (FF) survey which adopted a similar approach estimated food insecurity in UC at 43.6% [43]. While our values are still somewhat higher, it should be noted that the FF only made use of three of the USDA food security module questions, which would be likely to underestimate FIS compared with use of the full survey.

Our study group received between £135.58 and £300.67 per household per week depending on the number of adults and children. From this they were required to pay for housing, bills, outstanding debts and food. While gaining employment is considered the best route out of poverty, in work poverty remains due to low wages that don’t reflect the actual cost of living for plausible working hours [47]. Our data indicated that while the higher income levels attainable from employment may confer some protection, under current arrangements the rate at which benefits are withdrawn as wages rise means that those on low wages are not adequately protected from food insecurity. This remains a principal challenge for the UK income support policy [18]. For many, the income from low wages (including the current living wage) is insufficient to meet the expenditure requirements of a household [48]. The current study shows that, when income declines to a point at which a household becomes reliant on UC, then there is a roughly 85% chance that adults within that household will descend into food insecurity.

The few people in our sample who were food secure did not meet any specific criteria other than being more likely to be in work, in receipt of higher incomes, have no accommodation costs and not require extensive travel (> 15 min) to obtain food shopping. Comparison against the MIS at the time (Fig. 2) [41] indicated that people dependent on UC received around 40% of the amount deemed necessary to achieve a minimum basic standard of living, a situation which continues to be the case in 2024 [48]. We found that the proportion of MIS needed to reach 80% before half of the respondents were able to be food secure. To achieve this, the 279 people receiving below that threshold would require an increase in their income of on average £244.75 per week. The fact that those achieving the MIS were not universally FS suggests that additional challenges were faced by this group beyond achieving this level of income. It is very likely that debts which required servicing prior to expenditure on food would have been a major factor, but this was not measured in the current study.

Food insecurity resulted in a reduction of the quality and quantity of food consumed, with 84% unable to afford balanced meals and 43.6% not eating for entire days in the previous month. These figures represent a considerable increase from the low-income diet and nutrition survey where 36% said they could not afford to eat balanced meals and 5% were unable to eat for a whole day [49]. The level of food insecurity may be higher in our study population due to targeting of Universal Credit claimants at a time when the cost of living was increasing. The impact of such levels of food deprivation were apparent from the number of people recorded as underweight (6.4%) being 3 times greater than the national UK average (1.8%) [50]. The presence of children in the household did not appear to influence the likelihood of adults needing to starve themselves, however almost all (89%) of those with children who reported fasting for entire days sacrificed their own food to enable children to eat. Approximately half of those who starved for entire days resorted to buying food on credit, necessitating future sacrifices to satisfy their immediate survival requirements. This situation is increasingly documented in the national press ([51]).

The proportion recording intakes below the LRNI for various minerals (selenium, magnesium, zinc and calcium) was such that clinically apparent deficiencies, typically rare for western populations of adult women [52, 53], would be likely if reliance on UC continued for an extended period. Media reports are already appearing which support this [54–56]. While iron deficiency anaemia is observed in around 8% of UK women [57], the intakes recorded here show that more than 50% of UC recipients under 50 years consumed below the LRNI, implying that the proportion of this group exhibiting iron deficient anaemia is likely to be much higher than the national average. The clinical outcomes from such levels of deficiency are and will be extensive, resulting in early onset disease, increased prevalence and severity of infection, reproductive failure, extended proportion of life spent in ill health, followed by premature death [58]. It should be noted that the duration of reliance on UC is relatively extensive, particularly post-covid [59], with 5.7 million people on UC in July 2022. Around 25% of people spend more than 1 year out of work, so the size of the group exposed to an extended period of such nutrient deprivation would be considerable.

Considering the restricted nutrient intakes observed, with > 16% consuming no vegetables and almost 40% no fruit, the identification of very low food diversity was unsurprising. It was also noteworthy that contrary to what we saw for fruit and vegetable intake, the consumption of grains and in particular white bread, was significantly higher for UC recipients than all other groups. The energy derived from white bread (14.4%) was twice that seen in the typical adult population [60]. The reliance on bread and cereals reflects the need to purchase basic staples with a very limited income. This aligns with previous work showing food insecure populations consuming greater quantities of bread rolls than food secure [61]. Because of adoption of bread as a staple food, then this could potentially have resulted in a greater intake of calcium, niacin, thiamin and iron due to current fortification, although it was apparent that iron intake in the under 50s was still significantly lower in the BEANS compared with all NDNS groups. Folic acid intake may also be improved once its mandatory fortification into non whole meal flour comes into effect in late 2026. However, fortification of flour alone would be insufficient to overcome the numerous deficiencies seen in low income groups.

Lastly, the loss of the uplift resulted in an almost doubling of reported reliance on food banks. Previous studies have suggested that the introduction of the £20 week^− 1^ uplift to UC coincided with a reduction in the prevalence of poverty [62] and provided protection from food insecurity. The Trussell Trust findings saw that removal of the uplift to UC coincided with an increase in food bank usage while The Food Foundation food insecurity tracking in August 2021 reported 78% of households with a claim for Universal Credit felt that the removal of the £20 week^− 1^ uplift would make it harder for them to afford food [63]. This is reflected in our findings as an additional 21% of the population accessed a food bank after the removal (Supplementary Table 7).

There were several limitations to this study. We did not collect sufficient data prior to the loss of the uplift to effectively assess the material impact of its loss. Conclusions regarding food bank reliance are dependent on participant opinion and we were unable to compare nutrient consumption patterns consequent to the removal. The survey itself was extensive and detailed, which likely reduced the motivation for many to complete. Also, some participant responses were recorded in February, a period during which low-income households tend to struggle after the expense of Christmas, which may have influenced outcomes at that point. In addition, while response to the survey was robust, the number providing dietary data was limited and only available for females (18.5% of the study population) as such the results for males may have differed. We did not explore analyses according to plausible or non-plausible energy intake as the study focused on food insecurity and it was therefore reasonable to assume that for many, energy intakes would necessarily be low. Had we received more food diary data, this would have been carried out, but with the numbers we recruited, such analyses were not possible. Intake 24 is considered to provide a good approximation of intake and is adopted in the national data gathering for the NDNS. Whilst there is scope for variation, studies have indicated that it represents a generally reliable method [32, 64]. and uses a procedure found to be most acceptable to those on limited incomes [65]. To establish a clearer picture of the severity of the impact of reliance on UC on nutritional and health outcomes, a much larger study is necessary.

In conclusion, we find that reliance on UC during the period covering the end of 2021 to early 2022 almost guaranteed food insecurity, with nearly half of the population driven to starve for entire days. The driver of such desperate “decisions” was poverty. To maximise the number of UC recipients having enough money to feed themselves and their families, an income > 80% of the MIS would be necessary, but this would still only enable 50% to be food secure and therefore require additional interventions. The findings here are highly relevant to the current time when for some, food bank use has ceased to be regarded as a crisis measure and shows evidence of becoming a normalised part of the daily life on a low income [66]. We would expect that, after the rapid rise in food and utility prices since April 2022, the situation for people on UC today is even bleaker. Re-instating the £20 a week uplift and increasing benefit allowance in line with inflation would go some way to reducing the prevalence of food insecurity among Universal Credit claimants, as would removal of the necessity to wait for five weeks before receiving benefits.

## Electronic supplementary material

Below is the link to the electronic supplementary material.

Supplementary Material 1

Supplementary Material 2
