# Supplementary material for: Food insecurity amongst universal credit claimants: the benefits and nutrition study (BEANS), a cross-sectional online study
Source: Eur J Nutr. 2025 Mar 10;64(3):115. doi: 10.1007/s00394-025-03596-y (PMC11893655; doi:10.1007/s00394-025-03596-y)
Supplement: Supplementary file 1 — Supplementary Material 1 [file 394_2025_3596_MOESM1_ESM.pdf]

Food insecurity amongst Universal Credit claimants and nutritional security of females with a claim for Universal Credit: Benefits and Nutrition Study (BEANS), a cross sectional online study.

Michelle Thomas[1], Peter Rose[1], Lisa Coneyworth[1], Juliet Stone[2], Matt Padley[2], Patrick O'Reilly[3] and Simon Welham[1]

[1] School of Biosciences, Food Nutrition and Dietetics, University of Nottingham, Sutton Bonnington Campus, Nr Loughborough, LE12 5RD, UK
[2] Centre for Research in Social Policy, Loughborough University, Room U130 Brockington Building, Loughborough, Leicestershire LE11 3TU, UK
[3] School of humanities and social sciences, John Foster Building, Liverpool John Moores University, Liverpool, L3 5UZ
Corresponding Author: Dr Simon Welham
Email simon.welham@notttingham.ac.u

Supplementary Table 1 Number of servings or weight (g) of food in the adequacy category required to achieve maximum score of 5 based on energy intake groups.

	Food group			
	Vegetables	Fruits	Grain	Fibre
Energy (kcal)	Number of servings			Weight (g)
<1700	3	2	>= 6	20
>=1700- <2200	4	3	>= 9	25
>=2700	5	4	>= 11	30

Adapted from the scoring system as describe by Mariscal-Arcas et al [62]

Supplementary Table 2 Serving sizes based on BDA (British Dietetic Association) serving size guidelines (average taken for milk and milk products based on portion size for milk and yogurt)

DQI category		Food group	Serving size (g)	Half serving size (g)
Overall Variety	Within group variety	Meat	90	45
Overall Variety	Within group variety	Poultry	90	45
Overall Variety	Within group variety	Fish	140	70
Overall Variety	Within group variety	Eggs	120	60
Overall Variety	Within group variety	Dairy (Milk and milk products)	170	85
		Dairy (cheese)	30	15
Overall Variety	Within group variety	Dairy (Beans)	150	75
Overall Variety	Adequacy	Grains (Bread and cereal)	35	
Overall Variety	Adequacy	Grains (Rice and pasta)	150	
Overall Variety	Adequacy	Fruits and vegetables	80	

Variety within protein sources, values >= ½ a serving were considered as a meaningful quantity as per recommendations in Kim et al study.
Empty calorie foods: selected based on Eatwell guide for foods to be eaten less frequently.
NDNS data: Categories as laid out in the person level dietary data. Fruit recalculated to included fruit juice 100% to a maximum qty of 150ml

Supplementary Table 3 The proportion of respondents across each of the food security domains who are within one of Body Mass Index categories and their self-reported health.

	Total		High		Marginal		Low		Very low		P value	Food secure		Food insecure		P value
	**n**	**%**	**n**	**%**	**n**	**%**	**n**	**%**	**n**	**%**		**n**	**%**	**n**	**%**	
**BMI_groups**											0.654					0.762
Underweight	19	6.4	1	5.3	0	0.0	2	10.5	16	84.2		1	5.3	18	94.7	
Healthy weight	77	25.8	6	7.4	6	8.1	9	11.4	56	73.2		12	15.5	65	84.7	
Overweight	74	24.7	5	6.8	6	8.1	8	10.8	55	74.3		11	14.9	63	85.1	
Obesity	129	43.1	9	7.0	13	10.1	19	14.7	88	68.2		22	17.1	107	82.9	
**Total**	**299**															
**Health**											**0.008**					**<0.001**
Very good / good	89	27.3	13	14.6	11	12.4	11	12.4	54	60.7		24	27	65	73.0	
Fair	126	38.7	8	6.3	9	7.1	13	10.3	96	76.2		17	13	109	86.5	
Bad / Very Bad	111	34.0	3	2.7	5	4.5	12	10.8	91	82.0		8	7	103	92.8	
**Total**	**326**															

Supplementary Table 4 Estimated daily macronutrient intakes from diet only amongst females aged 23-61 years in BEANs and the NDNS year's 9-11 Equivalised household income tertiles.

	**BEANs** (n =43)			**EQV1 (n =202)**				**EQV 2 (n =202)**				**EQV 3 (n =249)**			
	**Median**	**Percentiles**		**Median**	**Percentiles**			**Median**	**Percentiles**			**Median**	**Percentiles**		
		**25**	75		25	75	**P value**		25	75	**P value**		25	75	**P value**
Total energy (kcal) diet only	1443	(1086)	(1723)	1520[a]	(1204)	(1771)	0.310	1546[a]	(1296)	(1799)	0.080	1660[b]	1330	1946	0.006
Protein (g) diet only	55.4	(39.1)	(78.3)	59.3[a]	(49.5)	(73.4)	0.331	66.0[a]	(52.0)	(79.0)	0.040	68.8[b]	58.5	82.2	0.002
Fat (g) diet only	51.4	(35.2)	(60.1)	57.6[a]	(39.8)	(71.7)	0.068	57.6[ab]	(43.8)	(71.5)	0.017	61.8[b]	47.1	79.7	<0.001
Saturated fatty acids (g) diet only	17.6	(13.4)	(25.8)	21.4	(13.9)	(27.6)	0.272	19.9	(14.8)	(27.5)	0.285	21.6	15.5	29.1	0.032
Carbohydrate (g) diet only	187.4	(142.6)	(230.7)	182.5	(145.1)	(217.9)	0.813	187.7	(145.5)	(224.4)	0.904	189.8	149.5	230.4	0.613

P values are comparison of BEANs to NDNS equivalised household income tertiles Mann Whitney

Superscript letter denotes significant differences between the NDNS Equivalised household income tertiles only.

Supplementary table 5 Comparison of energy and micronutrient intakes **(**percentage of the RNI and % below LRNI) amongst females aged 23-61 years with and income an income from Universal Credit (BEANS) and per the criteria for equivalised income tertiles in the National Diet and Nutrition Survey (years 9-11).

	**Beans (n = 43)**			**EQV 1 (n =202)**				**EQV 2 (n=202)**				**EQV 3 (n =249)**				
	**Median**	**25th**	**75th**	**Median**	**25th**	**75th**	**P value**	**Median**	**25th**	**75th**	**P value**	**Median**	**25th**	**75th**	**P value**	**P value (EQV's only)**
Energy (kcal)	1443	(1086)	(1723)	1520[a]	(1204)	(1771)	0.310	1546[a]	(1296)	(1799)	0.08	1660[b]	1330	1946	0.006	
	**n (total)**	**n**	**(%)**	**n (total)**	**n**	**(%)**	**P value**	**n (total)**	**n**	**(%)**	**P value**	**n (total)**	**n**	**(%)**	**P value**	
Underreportting [β]	38[Ŧ]	19	(50.0)	183[ŦŦ]	57	(31.1)	0.026	183[ŦŦ]	38	(20.8)	<0.001	233[ŦŦŦ]	50	(21.5)	<0.001	0.031

Ŧ five participants did not record a height or weight.

ŦŦ 19 participants did not record a height or weight.

ŦŦŦ 16 participants did not record height or weight.

Supplementary table 6 Estimated daily micronutrient intakes from diet only amongst females aged 23-61 years in BEANs and the NDNS year's 9-11 Equivalised household income tertiles.

	Beans (n =43)			Lowest (n =202) EQV 1				Middle (n=202) EQV 2				Highest (n =249) EQV 3			
	Median	Percentiles		Median	Percentiles		P value	Median	Percentiles		P value	Median	Percentiles		P value
		(25)	(75)		(25)	(75)			(25)	(75)			(25)	(75)	
Vitamin A (retinol equivalents) (µg)	410.8	(288.5)	(547.4)	564.6^{a}	(354.3)	(835.8)	0.003	656.6^{a}	(424.6)	(995.3)	<0.001	811.6^{b}	(497.1)	(1377.2)	<0.001
Thiamine (mg)	1.1	(0.9)	(1.6)	1.2^{a}	(0.9)	(1.5)	0.857	1.3^{b}	(1.1)	(1.6)	0.097	1.4^{c}	(1.1)	(1.7)	0.003
Riboflavin (mg)	1.2	(0.8)	(1.7)	1.2^{a}	(0.9)	(1.5)	0.639	1.3^{a}	(1.0)	(1.6)	0.198	1.5^{b}	(1.2)	(1.9)	0.001
Niacin equivalent (mg)	25.5	(18.5)	(35.8)	27.4^{a}	(21.9)	(34.0)	0.634	30.1^{ab}	(24.2)	(36.7)	0.116	31.6^{b}	(26.2)	(37.2)	0.023
Vitamin B6 (mg)	1.3	(0.9)	(1.7)	1.3^{a}	(1.1)	(1.6)	0.189	1.4^{a}	(1.1)	(1.8)	0.019	1.5^{b}	(1.2)	(1.8)	<0.001
Vitamin B12 (µg)	3.5	(2.0)	(4.9)	3.8^{a}	(2.6)	(5.1)	0.221	3.9^{ab}	(2.9)	(5.4)	0.040	4.5^{b}	(3.1)	(5.7)	0.004
Folate (µg)	169.3	(119.6)	(201.8)	172.4^{a}	(135.6)	(225.5)	0.161	189.5^{a}	(150.2)	(231.9)	0.006	219.1^{b}	(174.5)	(277.4)	<0.001
Vitamin C (mg)	43.5	(27.7)	(100)	50.0^{a}	(32.6)	(81.7)	0.522	64.0^{b}	(44.2)	(99.5)	0.019	80.9^{c}	(55.0)	(111.3)	<0.001
Sodium (mg)	1651.7	(1253.9)	(2092.4)	1696.5	(1347.2)	(2131.2)	0.815	1670.9	(1339.7)	(2178.4)	0.733	1862.9	(1408.5)	(2248.1)	0.252
Potassium (mg)	2199.3	(1710.7)	(2841.9)	2274.5^{a}	(1871.9)	(2709.7)	0.762	2483.2^{b}	(2030.3)	(3000.6)	0.034	2756.7^{c}	(2347.6)	(3282.9)	<0.001
Calcium (mg)	714.7	(490.5)	(956.9)	648.1^{a}	(496.9)	(849.6)	0.246	679.1^{a}	(522.4)	(856.6)	0.422	773.7^{b}	(609.4)	(945.5)	0.350
Magnesium (mg)	203.9	(158.6)	(276.3)	204.9^{a}	(164.8)	(251.9)	0.760	229.4^{b}	(185.6)	(277.6)	0.128	260.5^{c}	(217.1)	(313.3)	<0.001
Phosphorus (mg)	1002.9	(679.1)	(1358.0)	1022.1^{a}	(812.4)	(1243.3)	0.872	1090.1^{a}	(876.9)	(1297.0)	0.238	1170.2^{b}	(1002.1)	(1404.8)	0.009
Copper (mg)	0.9	(0.6)	(1.1)	0.9^{a}	(0.7)	(1.1)	0.294	1.0^{b}	(0.8)	(1.2)	0.010	1.1^{c}	(0.9)	(1.5)	0.000
Zinc (mg)	6.4	(4.7)	(8.8)	6.8^{a}	(5.4)	(8.2)	0.646	7.4^{a}	(5.9)	(8.9)	0.131	8.0^{b}	(6.4)	(9.7)	0.004
Iodine (µg)	104.2	(60.4)	(146.9)	111.8^{a}	(80.0)	(144.8)	0.211	124.4^{a}	(90.4)	(156.6)	0.018	140.4^{b}	(101.7)	(200.6)	<0.001
Selenium (µg)	27.38	(21.8)	(48.6)	37.9^{a}	(28.3)	(48.0)	0.021	40.1^{a}	(31.2)	(52.5)	<.001	45.5^{b}	(34.2)	(56.9)	<0.001
Iron (mg) <=50 years	7.9	(5.6)	(9.28)	8.5^{a}	(6.5)	(10.5)	0.193	9.2^{a}	(7.1)	(11.0)	0.024	10.2^{b}	(7.7)	(11.9)	<0.001
Iron (mg) >50 years	6.3	(4.0)	(11.1)	7.4^{a}	(5.3)	(9.4)	0.262	8.7^{b}	(7.3)	(10.2)	0.013	9.8^{b}	(8.1)	(11.9)	<0.001

P values are comparison of BEANs to NDNS equivalised household income tertiles.

Superscript letter denotes significant differences between the NDNS Equivalised household income tertiles only

<=50 years n = 36

>50 years n =7

Supplementary Table 7 Comparison of frequency and access of foodbank amongst BEANs participants before uprating was in place and after its removal.

	Total	
**Categories**	**n**	**(%)**
Not accessed the food bank before or after	135	(44.7)
Accessed foodbank before and after.	89	(29.5)
Accessed after food bank after but not before.	62	(20.5)
Accessed foodbank before but not after.	16	(5.3)
**Total**	**316**	**(100)**

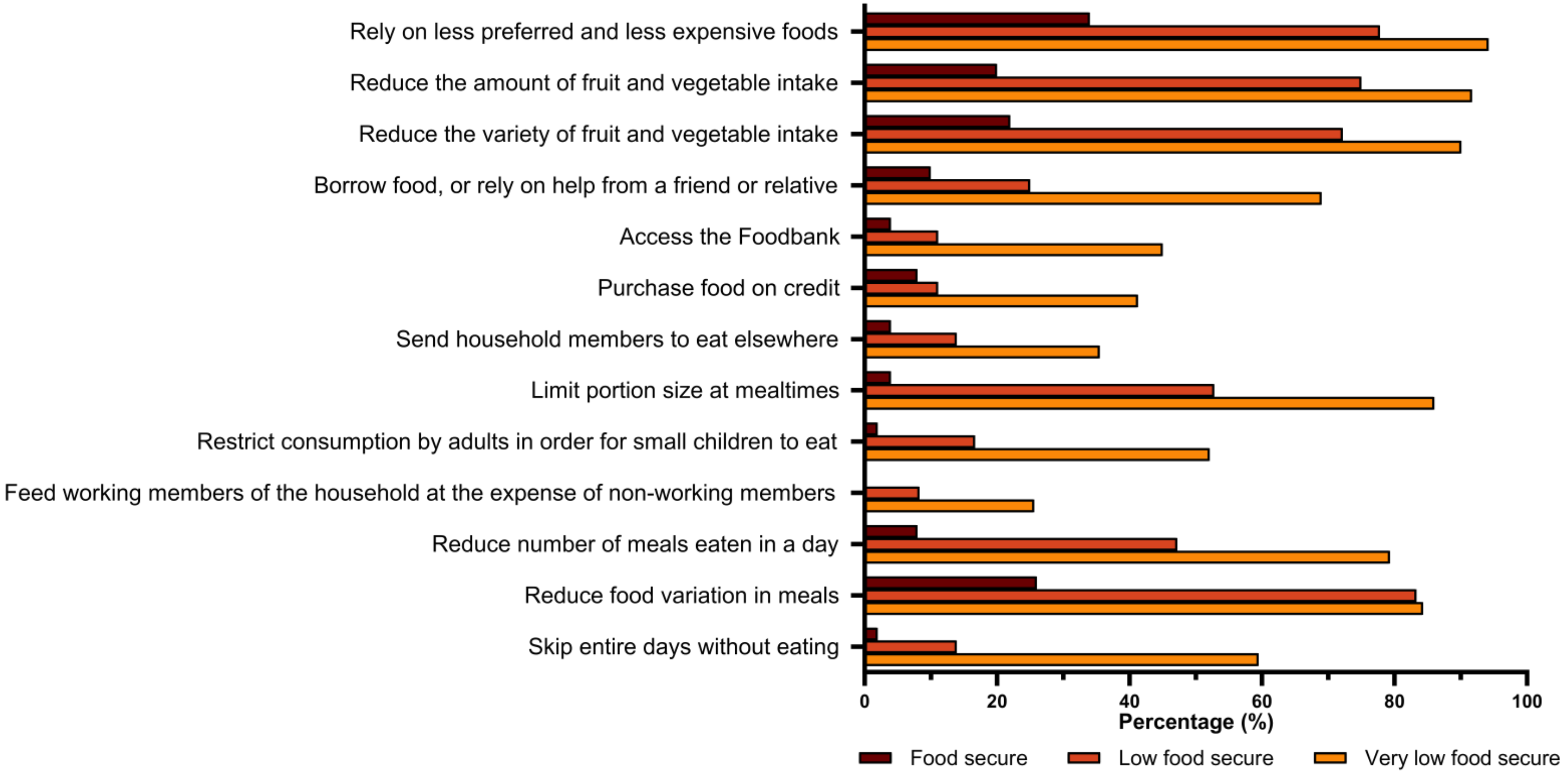

Supplementary Fig 1 Response rate for the type of coping strategies type used in the previous 7 days for food secure, low food secure and very low food secure.

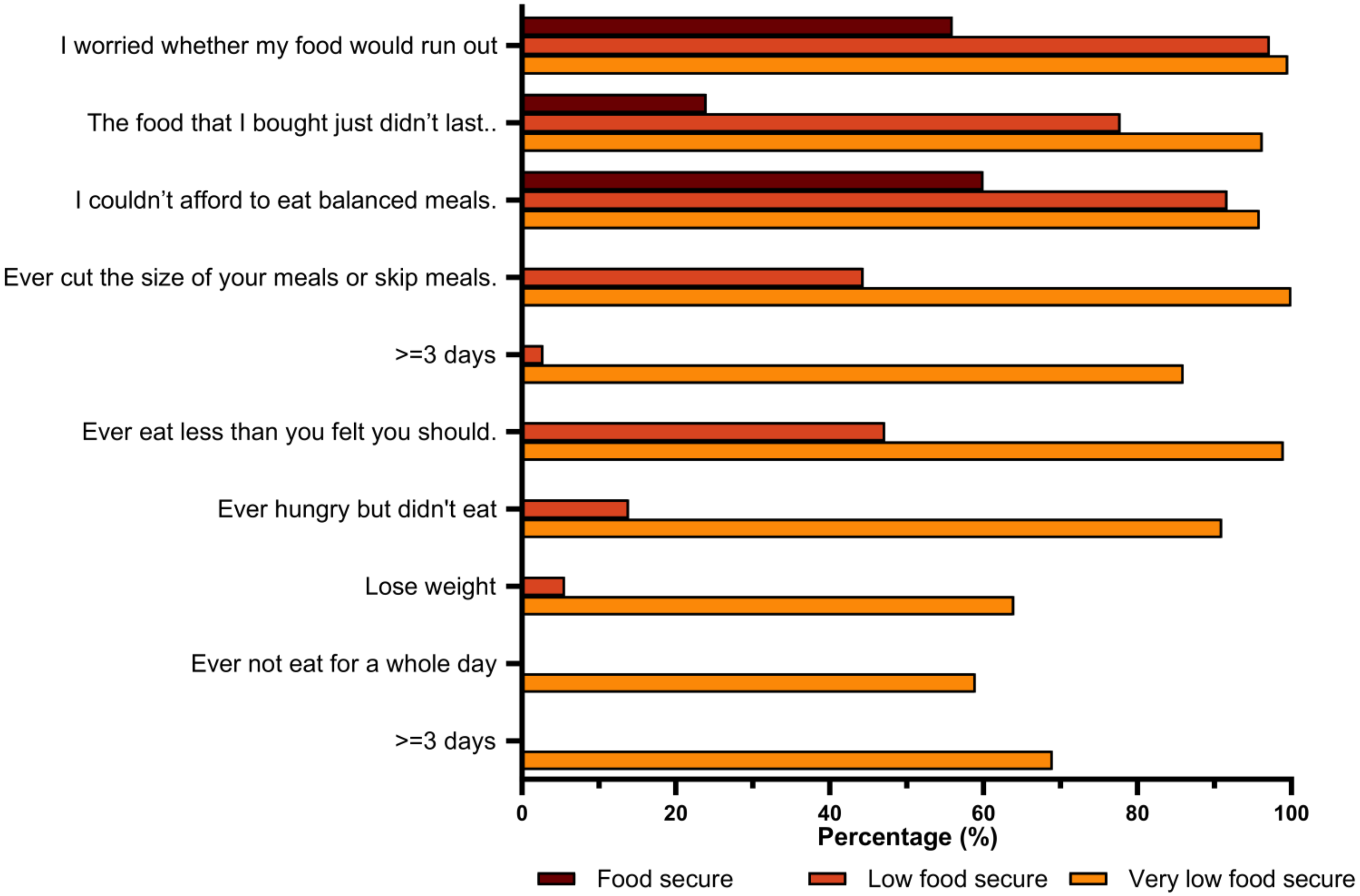

Supplementary Fig 2 Response rate for adult food security module split buy food secure, low food secure and very low food secure
