# Supplementary material for: Food insecurity amongst universal credit claimants: the benefits and nutrition study (BEANS), a cross-sectional online study
Source: Eur J Nutr. 2025 Mar 10;64(3):115. doi: 10.1007/s00394-025-03596-y (PMC11893655; doi:10.1007/s00394-025-03596-y)
Supplement: Supplementary file 2 — Supplementary Material 2 [file 394_2025_3596_MOESM2_ESM.pdf]

Universal Credit, Diet and Nutrition Study 2021

Participant Information

Welcome to the Universal Credit, Diet, and Nutrition Study

Thank you for your interest in our study, we would like to invite you to take part in our online survey which is looking into the influence of Universal Credit and the uplift on dietary intake and food security status of adults aged 18-65 years in the UK.

We ask you to complete one online survey, this will take no more than 25 minutes to complete. There is an option at the end of the survey to take part in a further study looking into types of foods consumed and their influence on micronutrient intakes, this is optional.

Please be assured any information you give us is confidential and we will anonymize all data so that no one will be able to identify you or any member of your family. We appreciate your interest in taking part in our study.

This research has been approved by the University of Nottingham Faculty of Medicine and Health Sciences Research Ethics Committee (Reference No: FMHS 318-0821)

If you have any questions about the study, please feel free to contact me on idns@nottingham.ac.uk.

Please find a link to the participant information sheet here:

https://static.onlinesurveys.ac.uk/media/account/171/survey/814466/question/participant_information_sheet_.docx

To ensure your responses are recorded please click on the finish button on the last page of the survey

Please can you tell us if you

1. Were you in receipt of the £20 uplift to Universal Credit? ✱ *Required*

- ◯ Yes
- ◯ No

2. Are aged between 18 - 65 years? ✱ *Required*

- ◯ Yes
- ◯ No

3. Consent to participating in this study? ✱ *Required*

- ◯ Yes
- ◯ No

Please tell us a little about yourself

4. What was your age on your last birthday (in years)? ✱ *Required*

5. What is you sex? ✱ *Required*

- Male
- Female
- Other
- Prefer not to say

6. Are you currently ✱ *Required*

- Married and living with your partner
- A civil partner in a legally – recognized Civil Partnership
- Living with your partner
- Single (never been married)
- Married and separated from your partner
- Divorced
- Widowed

7. To which of these groups do you consider you belong? ✱ *Required*

Please tell us a little bit about your household

8. What is your postcode please? ✱ *Required*

9. Please indicate in which ways you occupy your accommodation ✱ *Required*

10. How many adults aged over 18 years including yourself live in your household? ✱ *Required*

- 1
- 2
- 3
- 4
- more than 5

11. Do you have children living with you in your household? ✱ *Required*

- Yes
- No
- prefer not to say

Please tell us a little bit about the children living in your household

12. How many children live with you in your household? (Please select age group and number of children)

	Number of children						
	0	1	2	3	4	5	6 or more
Age 0-1 years	○	○	○	○	○	○	○
Age 2-4 years	○	○	○	○	○	○	○
Age 5-7 years	○	○	○	○	○	○	○
Age 8-10 years	○	○	○	○	○	○	○
Age 11-12 years	○	○	○	○	○	○	○
Age 13-15 years	○	○	○	○	○	○	○
Age 16-18 years	○	○	○	○	○	○	○

13. How many children living in your household are dependent on you financially? ✱ *Required*

- ○ 0
- ○ 1
- ○ 2
- ○ 3
- ○ 4
- ○ 5
- ○ 6 or more
- ○ Not applicable

14. Are you pregnant or breast feeding? *Optional*

- ○ Yes
- ○ No
- ○ Not applicable
- ○ Prefer not to say

15. Is/are your child/ren entitled to Free School Meals ✱ *Required*

- ○ Yes
- ○ No

- Prefer not to say
- Not applicable

Check if your child is eligible for free school meals in England and apply here https://www.gov.uk/apply-free-school-meals

16. Are you in receipt of Healthy Start Vouchers? ✱ *Required*

- Yes
- No
- Prefer not to say
- Not applicable

Check if you are eligible and apply for Healthy Start Vouchers here https://www.healthystart.nhs.uk/how-to-apply/

I would now like to ask you a couple of questions about education and work-related training

17. At what age did you finish your full-time education at school or college? ✱ *Required*

____

18. Do you have any qualifications (e.g. from school, college, university, or from work or government schemes?) ✱ *Required*

- Yes
- No
- Prefer not to say

18.a. If yes, please tell me if you have any of the qualifications listed, please look at the list and tell me the first one you come to that you have passed

____

Employment

19. **Please may you tell me your employement status** ✱ *Required*

19.a. If you selected Other, please specify:

20. If your are in full or part time employment: How many hours do you work per week in your job? ✱ *Required*

- 0-8
- 9-16
- 17-30
- 31-40
- More than 40 hours
- Not applicable

21. Are you an employee or self employed? ✱ *Required*

- Employee
- Self-Employed
- Not applicable

22. Please may you tell me what is your main job (e.g. cashier, nursery nurse, engineer, teacher, chef, cook, builder, retail assistant or leave blank if prefer not to say) *Optional*

Income source

23. Please could you tell me which kinds of income you *(and your husband, wife partner)* receive? ✱ *Required*

- ☐ Earnings from employment or self-employment
- ☐ State retirement pension
- ☐ Pension from former employer
- ☐ Personal Pensions
- ☐ Job-Seekers Allowance
- ☐ Employment and Support Allowance
- ☐ Income Support
- ☐ Pension Credit
- ☐ Working Tax Credit
- ☐ Child Tax Credit
- ☐ Housing benefit
- ☐ Universal Credit
- ☐ Other

23.a. If you selected Other, please specify:

24. Please could you tell me what your average weekly household net income is (£) (after tax and deductions) ✱ *Required*

Food purchase and shopping habits

25. From the list below, please select the top three Supermarkets you shop at most frequently. On a score of 1-3 please indicate the shop you use most frequently (1 = most frequent, 3 = least frequent) ✱ *Required*

Please don't select more than 1 answer(s) per row.

Please select between 1 and 3 answers.

Please don't select more than 1 answer(s) in any single column.

	1	2	3
Tesco	☐	☐	☐
Morrisons	☐	☐	☐
M & S Food	☐	☐	☐
Lidl	☐	☐	☐
Sainsbury	☐	☐	☐
Aldi	☐	☐	☐
Tesco Express	☐	☐	☐
Asda	☐	☐	☐
The Co-operative Food	☐	☐	☐
Iceland	☐	☐	☐
Waitrose	☐	☐	☐
The Co-operative	☐	☐	☐
Costco	☐	☐	☐
Farmfoods	☐	☐	☐
SPAR	☐	☐	☐
Ocado	☐	☐	☐
Costcutter	☐	☐	☐
Nisa	☐	☐	☐
One Stop	☐	☐	☐
Premier Stores	☐	☐	☐
Londis	☐	☐	☐
Other	☐	☐	☐

25.a. Is the supermarket you selected as 1, where you do your main shop? ✱ *Required*

- ○ Yes
- ○ No

25.a.i. If No please tell us where you do your main shop

25.a.ii. Do you use a supermarket loyalty card for your main shop? ✱ Required

- Yes
- No
- Prefer not to say

25.a.ii.a. If yes, may we ask if you would be willing to share your food purchase data from your loyalty card?

- Yes
- No

25.a.ii.a.i. If, yes please enter your loyalty card number found on your card or supermarket App. This will be used to understand food purchases, cost and estimate nutrients available to the household.

25.b. How often do you shop at your main supermarket?

- Once a month
- 2-3 times a month
- Once a week
- 2-3 times a week
- 4-6 times a week
- Once a day
- More than once a day

26. Thinking about your main shop do you purchase fruit and vegetables ✱ Required

- Yes
- No

26.a. If yes, How often do you purchase them?

- less than once a month
- 1-3 per month
- Once a week

- 2-4 per week
- 5-6 per week
- once a day

26.a.i. If yes, On average how much do you spend (£) on fruit and vegetables each time you shop?

- 0-5
- 6-10
- 11-15
- 16-20
- more than £20
- Don't know

26.a.i.a. Please tell us what type of fruit and vegetables you purchase most frequently (optional)

26.b. If No, Do you purchase fruit and vegetables elsewhere?

- Yes
- No

26.b.i. If Yes, where do you purchase them?

26.b.ii. If yes, How often do you purchase them?

- Less than once a month
- 1-3 per month
- Once a week
- 2-4 per week
- 5-6 per week
- Once a day

26.b.ii.a. If yes, On average how much do you spend (£) on fruit and vegetables each time you shop?

- 0-5

- 6-10
- 11-15
- 16-20
- more than £20
- Don't know

26.b.ii.b. Please tell us what type of fruit and vegetables you purchase most frequently (optional)

27. Thinking about your main shop do you purchase beans, pulses, fish, eggs, meat and other protein products?
✱ *Required*

- Yes
- No

27.a. If yes, How often do you purchase them?

- less than once a month
- 1-3 per month
- Once a week
- 2-4 per week
- 5-6 per week
- once a day

27.a.i. If yes, On average how much do you spend (£) on beans, pulses, fish, eggs, meat and other protein each time you shop?

- 0-5
- 6-10
- 11-15
- 16-20
- more than £20
- Don't know

27.a.i.a. Please tell us what type of beans, pulses, fish, eggs, meat and other protein products you purchase most frequently (optional)

27.b. If No, Do you purchase beans, pulses, fish, eggs, meat and other protein products elsewhere?

- Yes
- No

27.b.i. If Yes, where do you purchase them?

27.b.ii. If yes, How often do you purchase them?

- Less than once a month
- 1-3 per month
- Once a week
- 2-4 per week
- 5-6 per week
- Once a day

27.b.ii.a. If yes, On average how much do you spend (£) on beans, pulses, fish, eggs, meat and other protein products each time you shop?

- 0-5
- 6-10
- 11-15
- 16-20
- more than £20
- Don't know

27.b.ii.b. Please tell us what type of beans, pulses, fish, eggs, meat and other protein products you purchase most frequently (optional)

28. Thinking about your main shop do you purchase Dairy and Dairy alternatives? (e.g milk, soya milk, cheese) ✱

Required

- Yes
- No

28.a. If yes, How often do you purchase them?

- less than once a month
- 1-3 per month
- Once a week
- 2-4 per week
- 5-6 per week
- once a day

28.a.i. If yes, On average how much do you spend (£) on Dairy or Dairy Alternatives each time you shop?

- 0-5
- 6-10
- 11-15
- 16-20
- more than £20
- Don't know

28.a.i.a. Please tell us what type of Dairy or Dairy Alternatives you purchase most frequently (optional)

28.b. If No, Do you purchase Dairy or Dairy Alternatives elsewhere?

- Yes
- No

28.b.i. If Yes, where do you purchase them?

28.b.ii. If yes, How often do you purchase them?

- Less than once a month
- 1-3 per month
- Once a week
- 2-4 per week
- 5-6 per week
- Once a day

28.b.ii.a. If yes, On average how much do you spend (£) on Dairy or Dairy Alternatives products each time you shop?

- 0-5
- 6-10
- 11-15
- 16-20
- more than £20
- Don't know

28.b.ii.b. Please tell us what type of Dairy or Dairy Alternatives you purchase most frequently (optional)

29. Thinking about your main shop do you purchase convenience products (e.g. Ready meals, pasta sauces, pizza, chips, pot noodles and readymade sandwich fillings?) ✱ *Required*

- Yes
- No

29.a. If yes, How often do you purchase them?

- less than once a month
- 1-3 per month
- Once a week
- 2-4 per week
- 5-6 per week
- once a day

29.a.i. If yes, On average how much do you spend (£) convenience products (e.g. Ready meals, pasta sauces,

pizza, chips, pot noodles and readymade sandwich fillings?)

- 0-5
- 6-10
- 11-15
- 16-20
- more than £20
- Don't know

29.a.i.a. Please tell us what type of convenience products (e.g. Ready meals, pasta sauces, pizza, chips, pot noodles and readymade sandwich fillings?) (optional)

29.b. If No, do you purchase convenience products elsewhere? (e.g. Ready meals, pasta sauces, pizza, chips, pot noodles and readymade sandwich fillings)

- Yes
- No

29.b.i. If Yes, where do you purchase them?

29.b.ii. If yes, How often do you purchase them?

- Less than once a month
- 1-3 per month
- Once a week
- 2-4 per week
- 5-6 per week
- Once a day

29.b.ii.a. If yes, On average how much do you spend (£) on crisps, cakes, biscuits, chocolate or sweets or any other sweet or savoury snacks products each time you shop?

- 0-5
- 6-10
- 11-15

- 16-20
- more than £20
- Don't know

29.b.ii.b. Please tell us what type of crisps, cakes, biscuits, chocolate or sweets or any other sweet or savoury snacks you purchase most frequently (optional)

30. Thinking about your main shop do you purchase crisps, cakes, biscuits, chocolate or sweets or any other sweet or savoury snacks? ✱ *Required*

- Yes
- No

30.a. If yes, How often do you purchase them?

- less than once a month
- 1-3 per month
- Once a week
- 2-4 per week
- 5-6 per week
- once a day

30.a.i. If yes, On average how much do you spend (£) on crisps, cakes, biscuits, chocolate or sweets or any other sweet or savoury snacks each time you shop?

- 0-5
- 6-10
- 11-15
- 16-20
- more than £20
- Don't know

30.a.i.a. Please tell us what type of crisps, cakes, biscuits, chocolate or sweets or any other sweet or savoury snacks you purchase most frequently (optional)

30.b. If No, Do you purchase crisps, cakes, biscuits, chocolate or sweets or any other sweet or savoury snacks elsewhere?

- Yes
- No

30.b.i. If Yes, where do you purchase them?

30.b.ii. If yes, How often do you purchase them?

- Less than once a month
- 1-3 per month
- Once a week
- 2-4 per week
- 5-6 per week
- Once a day

30.b.ii.a. If yes, On average how much do you spend (£) on crisps, cakes, biscuits, chocolate or sweets or any other sweet or savoury snacks products each time you shop?

- 0-5
- 6-10
- 11-15
- 16-20
- more than £20
- Don't know

30.b.ii.b. Please tell us what type of crisps, cakes, biscuits, chocolate or sweets or any other sweet or savoury snacks you purchase most frequently (optional)

31. What is the main reason why you shop at your main shop (please select all that apply)

	Main reason for why you shop at your main shop
Cheapest	☐
Range of goods	☐
Easiest to get to	☐
Closest	☐
Most convenient	☐
Shopping facility less than 5 minutes away	☐

32. How long does it take to get there and home again, not including the time spent doing the shopping? ✱ *Required*

- ○ Less than 15 minutes
- ○ 15 minutes, less than 30
- ○ 30 minutes, less than 1 hour
- ○ 1 hour, less than 1½ hours
- ○ 1½ hours, less than 2 hours
- ○ 2 hours or more

33. How do you/they usually get the food home? ✱ *Required*

- ○ By bus
- ○ By car
- ○ By taxi
- ○ By train
- ○ By bicycle
- ○ Home delivery
- ○ By other transport

34. Could you tell me how much you (your household) usually spends on food and drink each week? Please include main shopping, top-up shopping, school dinners and money given to children for food purchases as well as money spent on food bought from cafes and restaurants. Please do not include alcoholic drinks or other items such as cleaning materials, cigarettes, pet food, newspapers or magazines ✱ *Required*

- ○ 0 - 30
- ○ 31 - 50

- 51 - 70
- 71 - 80
- 81 - 100
- 101-120
- 121 – 140
- 141 – 150
- 151 – 160
- 161 – 180
- 181 – 200
- 201 – 220
- 221 – 240
- 241 – 260
- 261 – 280
- 281 – 300
- 301 – 320
- 321 – 340
- 341 – 360
- 361 – 380
- 381 – 400
- 401 – 450
- more than £450 per week

35. How often do you go food shopping (inculding your main shop and top up shopping ?) ✱ *Required*

- Once a month
- 1-3 times a month
- Once a week
- 2-4 time per week
- 5-6 times per week
- Once a day
- More than once a day

36. Please indicate if you

	Frequency				
	Always	Almost always	Often	Seldom	Never
Prepare a shopping list before you go shopping	○	○	○	○	○

Have a regular shopping list	○	○	○	○	○
Buy branded products?	○	○	○	○	○
Buy own brand products?	○	○	○	○	○
Buy yellow ticketed items (discounted)	○	○	○	○	○

37. In your own words can you please tell me how your income influences the foods you buy and your diet?

38. Before the £20 uplift to Universal Credit did you access food from a Foodbank or other charity organisation?

- ○ Yes
- ○ No
- ○ Prefer not to say

39. Since the removal of the £20 uplift to universal Credit have you accessed food from a Foodbank or other charity organisation?

- ○ Yes
- ○ No
- ○ Prefer not to say

40. During the COVID-19 pandemic, did you experience any of these situations preventing you from obtaining food? (please select all that apply) ✱ *Required*

- ☐ The shops did not have the food we needed
- ☐ Loss of income/ employment
- ☐ Low income: not enough money for food
- ☐ Self-isolation: I could not go out and did not have any other way to get the food you needed
- ☐ For other reasons
- ☐ Prefer not to answer
- ☐ None of the above

41. Since the **easing of restrictions** associated with COVID-19 pandemic, did you experience any of these situations preventing you from obtaining food? (please select all that apply) ✱ *Required*

- ☐ The shops did not have the food we needed
- ☐ Loss of income/ employment
- ☐ Low income: not enough money for food
- ☐ Self-isolation: I could not go out and did not have any other way to get the food you needed
- ☐ For other reasons
- ☐ Prefer not to answer
- ☐ None of the above

Influence of the £20 uplift to Universal Credit on food purchase

42. How do you think the £20 uplift to Universal Credit influenced your FRUIT and VEGETABLE intake? ✱ *Required*

- Increased
- Stayed the same
- Decreased

43. How do you think the £20 uplift to Universal Credit influenced your MEAT and MEAT PRODUCT intake? ✱ *Required*

- Increased
- Stayed the same
- Decreased

44. How do you think the £20 uplift to Universal Credit influenced your FISH and SEAFOOD intake? ✱ *Required*

- Increased
- Stayed the same
- Decreased

45. How do you think the £20 uplift to Universal Credit influenced your MILK and DAIRY intake? ✱ *Required*

- Increased
- Stayed the same
- Decreased

46. How do you think the £20 uplift to Universal Credit influenced your CEREAL and CEREAL PRODUCT intake? (e.g, pasta, rice, pizza, breakfast cereal, bread)

- Increased
- Stayed the same
- Decreased

47. How do you think the £20 uplift to Universal Credit influenced your SNACK and SNACK PRODUCT intake? (e.g, crisps, sweets, chocolate)

- Increased
- Stayed the same
- Decreased

Influence of the removal of the £20 uplift to Universal Credit on food purchase

48. How do you think the removal of the £20 uplift to Universal Credit influenced your FRUIT and VEGETABLE intake? ✱ *Required*

- Increased
- Stayed the same
- Decreased

49. How do you think the removal of the £20 uplift to Universal Credit influenced your MEAT and MEAT PRODUCT intake? ✱ *Required*

- Increased
- Stayed the same
- Decreased

50. How do you think the removal of the £20 uplift to Universal Credit influenced your FISH and SEAFOOD intake? ✱ *Required*

- Increased
- Stayed the same
- Decreased

51. How do you think the removal of the £20 uplift to Universal Credit influenced your MILK and DAIRY intake? ✱ *Required*

- Increased
- Stayed the same
- Decreased

52. How do you think the removal of the £20 uplift to Universal Credit influenced your CEREAL and CEREAL PRODUCT intake? (e.g, pasta, rice, pizza, breakfast cereal, bread)

- Increased
- Stayed the same

- Decreased

53. How do you think the removal of the £20 uplift to Universal Credit influenced your SNACK and SNACK PRODUCT intake? (e.g, crisps, sweets, chocolate)

- Increased
- Stayed the same
- Decreased

These next questions are about the food eaten in your household in the last 30 days, since the time of completing this survey and whether you were able to afford the food you need

54. I worried whether my food would run out before I got money to buy more. Was that often true, sometimes true, or never true for (you/your household) in the last 30 days? ✱ *Required*

- Often true
- Sometimes true
- Never true
- Prefer not to say

54.a. The food that I bought just didn't last, and I didn't have money to get more. Was that often, sometimes, or never true for (you/your household) in the last 30 days? ✱ *Required*

- Often true
- Sometimes true
- Never true
- Prefer not to say

54.b. I couldn't afford to eat balanced meals. Was that often, sometimes, or never true for (you/your household) in the last 30 days? ✱ *Required*

- Often true
- Sometimes true
- Never true
- Prefer not to say

55. In the last 30 days, did (you/you or other adults in your household) ever cut the size of your meals or skip meals because there wasn't enough money for food? ✱ *Required*

- Yes
- No
- Prefer not to say

55.a. If yes: How often did this happen—almost every day, some days but not every day, or only 1 or 2 days?

- Almost every day
- Some days but not every day

- Only 1 or 2 days

56. In the last 30 days, did you ever eat less than you felt you should because there wasn't enough money for food? ✱ *Required*

- Yes
- No
- Prefer not to say

57. In the last 30 days, were you every hungry but didn't eat because there wasn't enough money for food? ✱ *Required*

- Yes
- No
- Prefer not to say

58. In the last 30 days, did you lose weight because there wasn't enough money for food? ✱ *Required*

- Yes
- No
- Prefer not to say

59. In the last 30 days did (you/you or other adults in your household) ever not eat for a whole day because there wasn't enough money for food? ✱ *Required*

- Yes
- No
- Prefer not to say

59.a. If yes: How often did this happen—almost every day, some days but not every day, or in only 1 or 2 days?

- Almost every day
- Some days but not every day
- Only 1 or 2 day

If you are experiencing food insecurity the Food Bank are a community organisation providing emergency food to people in crisis. Please access the link here to find your nearest food bank https://www.trusselltrust.org/get-help/find-a-foodbank/

Coping strategies

60. In the past 7 days, if there have been times when you did not have enough food or money to buy food, how many days has your household had to:

	Number of days ✱ Required								
	1	2	3	4	5	6	7	None	Not applicable
Rely on less preferred and less expensive foods?	◯	◯	◯	◯	◯	◯	◯	◯	◯
Reduce the amount of fruit and vegetable intake?	◯	◯	◯	◯	◯	◯	◯	◯	◯
Reduce the variety of fruit and vegetable intake?	◯	◯	◯	◯	◯	◯	◯	◯	◯
Borrow food, or rely on help from a friend or relative?	◯	◯	◯	◯	◯	◯	◯	◯	◯
Access the Food Bank	◯	◯	◯	◯	◯	◯	◯	◯	◯
Purchase food on credit?	◯	◯	◯	◯	◯	◯	◯	◯	◯
Send household members to eat elsewhere?	◯	◯	◯	◯	◯	◯	◯	◯	◯
Limit portion size at mealtimes?	◯	◯	◯	◯	◯	◯	◯	◯	◯
Restrict consumption by adults in order for small children to eat?	◯	◯	◯	◯	◯	◯	◯	◯	◯
Feed working members of the household at the expense of non-working members?	◯	◯	◯	◯	◯	◯	◯	◯	◯
Reduce number of meals eaten in a day?	◯	◯	◯	◯	◯	◯	◯	◯	◯
Reduce food variation in meals? (eat the same foods at each meal occasion or eat the same foods for consecutive days)	◯	◯	◯	◯	◯	◯	◯	◯	◯
Skip entire days without eating?	◯	◯	◯	◯	◯	◯	◯	◯	◯

Food and Cooking Skills

61. "Please tell us which of the following you do (or use) and "On a scale from 1 to 7 where 1 means very poor and 7 means very good , please say how good you are at ✱ *Required*

Please don't select more than 1 answer(s) per row.

Please select at least 8 answer(s).

	1	2	3	4	4	5	6	7	Do not do/use
'Chop, mix and stir foods, for example chopping vegetables, dicing an onion, cubing meat, mixing and stirring food together in a pot/bowl	☐	☐	☐	☐	☐	☐	☐	☐	☐
'Blend foods to make them smooth, like soups or sauces' (using a whisk/blender/food processor etc.)	☐	☐	☐	☐	☐	☐	☐	☐	☐
Steam food (where the food doesn't touch the water but gets cooked by the steam)	☐	☐	☐	☐	☐	☐	☐	☐	☐
Boil or simmer food (cooking it in a pan of hot, boiling/bubbling water)	☐	☐	☐	☐	☐	☐	☐	☐	☐
Stew food (cooking it for a long time (usually more than an hour) in a liquid or sauce at a medium heat, not boiling) e.g. beef stew	☐	☐	☐	☐	☐	☐	☐	☐	☐
Roast food in the oven, for example raw meat/chicken, fish, vegetables etc	☐	☐	☐	☐	☐	☐	☐	☐	☐

Fry/stir-fry food in a frying pan/wok with oil or fat using the hob/ gas rings/hot plates	☐	☐	☐	☐	☐	☐	☐	☐	☐
Microwave food (not drinks/liquid) including heating ready-meals	☐	☐	☐	☐	☐	☐	☐	☐	☐

62. "Please tell us which of the following you do (or use) and "On a scale from 1 to 7 where 1 means very poor and 7 means very good , please say how good you are at ✱ *Required*

Please don't select more than 1 answer(s) per row.

Please select at least 6 answer(s).

	1	2	3	4	5	6	7	Do not do/use
Bake goods such as cakes, buns, cupcakes, scones, bread etc., using basic/raw ingredients or mixes	☐	☐	☐	☐	☐	☐	☐	☐
Peel and chop vegetables (including potatoes, carrots, onions, broccoli)	☐	☐	☐	☐	☐	☐	☐	☐
Prepare and cook raw meat/poultry	☐	☐	☐	☐	☐	☐	☐	☐
Prepare and cook raw fish	☐	☐	☐	☐	☐	☐	☐	☐
Make sauces and gravy from scratch (no ready-made jars, pastes or granules)	☐	☐	☐	☐	☐	☐	☐	☐
Use herbs and spices to flavour dishes	☐	☐	☐	☐	☐	☐	☐	☐

63. "Please tell us which of the following you do (or use) and "On a scale from 1 to 7 where 1 means very poor and 7 means very good , please say how good you are at ✱ *Required*

Please don't select more than 1 answer(s) per row.

Please select at least 10 answer(s).

	1	2	3	4	5	6	7	Do not do/use
plan meals ahead? (e.g. for the day/week ahead)	☐	☐	☐	☐	☐	☐	☐	☐
prepare meals in advance? e.g. packed lunch, partly preparing a meal in advance	☐	☐	☐	☐	☐	☐	☐	☐
follow recipes when cooking?	☐	☐	☐	☐	☐	☐	☐	☐
shop with a grocery list?	☐	☐	☐	☐	☐	☐	☐	☐
shop with specific meals in mind?	☐	☐	☐	☐	☐	☐	☐	☐
plan how much food to buy?	☐	☐	☐	☐	☐	☐	☐	☐
compare prices before you buy food?	☐	☐	☐	☐	☐	☐	☐	☐
know what budget you have to spend on food?	☐	☐	☐	☐	☐	☐	☐	☐
buy food in season to save money?	☐	☐	☐	☐	☐	☐	☐	☐
buy cheaper cuts of meat to save money?	☐	☐	☐	☐	☐	☐	☐	☐

64. “Please tell us which of the following you do (or use) and “On a scale from 1 to 7 where 1 means very poor and 7 means very good , please say how good you are at ✱ *Required*

Please don't select more than 1 answer(s) per row.

Please select at least 9 answer(s).

	1	2	3	4	5	6	7	Do not do/ use
cook more or double recipes which can be used for another meal?	☐	☐	☐	☐	☐	☐	☐	☐

prepare or cook a healthy meal with only few ingredients on hand?	☐	☐	☐	☐	☐	☐	☐	☐
prepare or cook a meal with limited time?	☐	☐	☐	☐	☐	☐	☐	☐
use leftovers to create another meal?	☐	☐	☐	☐	☐	☐	☐	☐
keep basic items in your cupboard for putting meals together? e.g. herbs/spices, dried/tinned goods?	☐	☐	☐	☐	☐	☐	☐	☐
read the best-before date on food?	☐	☐	☐	☐	☐	☐	☐	☐
read the storage and use-by information on food packets?	☐	☐	☐	☐	☐	☐	☐	☐
read the nutrition information on food labels?	☐	☐	☐	☐	☐	☐	☐	☐
balance meals based on nutrition advice on what is healthy?	☐	☐	☐	☐	☐	☐	☐	☐

Health

Please may you tell me your weight in either kilograms (kg), stones and pounds (st lbs) or pounds (lbs)

65. Kilograms

66. Stones and pounds

67. Pounds

Please may you tell me your height in either feet and inches (ft in) or meters (m) or centimeters (cm's)

68. Feet and Inches

69. Meters

70. Centimeters

71. How is your health in general? Would you say it was

- ◯ Very good
- ◯ Good
- ◯ Fair
- ◯ Bad
- ◯ Very bad

Next stage of the study

72. Would you like to take part in a further study looking into diet and micronutrient intakes? The study is online and involves the completion of three or four 24-hour dietary recalls ✱ *Required*

- ○ Yes
- ○ No

72.a. If yes, please enter your email address for us to contact you

[ ]

73. Do you live in the Nottingham area and would you like to participate in the basic cooking skills online sessions?

[ ]

73.a. If yes, please enter your email address for us to contact you

[ ]

74. Are you a customer of the Henley Grub Hub?

- ○ Yes
- ○ No

74.a. If yes and you would like to receive a voucher for one weeks subscription to the Grub Hub, please enter your email address

[ ]

Research funded by

Thank you for taking part in our study, your help is greatly appreciated.

Key for selection options

7 - To which of these groups do you consider you belong?

- English/Welsh/Scottish/Northern Irish/British
- Irish
- Gypsy of Irish Traveller
- Any other white background, please describe
- White and Black Caribbean
- White and Black African
- White and Asian
- Any other mixed/multiple ethnic background, please describe
- Indian
- Pakistani
- Bangladeshi
- Chinese
- Any other Asian background, please describe
- African
- Caribbean
- Any other Black/African/Caribbean background, please describe
- Arab
- Other ethnic group

9 - Please indicate in which ways you occupy your accommodation

- Own (own outright no mortgage)
- Mortgage (buying it with the help of a mortgage or loan)
- Shared ownership (part rent and part mortgage)
- Rent (Private landlord)
- Rent (Council/local authority)
- Rent (Housing Association, charitable trust)
- Rent (Relative or friend)
- Rent (Employer)
- Live here rent free
- Other (please specify)

17 - At what age did you finish your full-time education at school or college?

Not finished yet
Never went to school
14 or under
15
16
17
18
19 or over

18.a - If yes, please tell me if you have any of the qualifications listed, please look at the list and tell me the first one you come to that you have passed

Higher degree, e.g. MSc, MA, MBA, PGCE, PhD
Level 5 NVQ / SVQ
BTEC Advanced Professional Diploma/Certificate
First degree, e.g. BSc, BA, BEd, MA at first degree level
Level 4 NVQ / SVQ
HNC / HND
BTEC Higher National or Professional Diploma/Certificate
A-level
As-level
Level 3 NVQ / SVQ
ONC / OND
BTEC Advanced or National Diploma/Certificate
City & Guilds Advanced Craft / Part 3
Advanced GNVQ; Vocational A Level
Advanced Modern Apprenticeship
GCSE grade A*-C
Level 2 NVQ / SVQ
BTEC Intermediate or First Diploma/Certificate
City & Guilds Craft / Part 2
Intermediate GNVQ
GCSE grade D-G
Level 1 NVQ / SVQ
BTEC Foundation or Introductory Diploma/Certificate
Other qualifications

19 - Please may you tell me your employement status

Going to school, college, or university full-time
Going to school, college or university and working
In full or part-time employment
Not working at the moment
Other

24 - Please could you tell me what your average weekly household net income is (£) (after tax and deductions)

0 – 120
121 – 140
141 – 150
151 – 160
161 – 180

- 181 – 200
- 201 – 220
- 221 – 240
- 241 – 260
- 261 – 280
- 281 – 300
- 301 – 320
- 321 – 340
- 341 – 360
- 361 – 380
- 381 – 400
- 401 – 450
- 451 – 500
- 501 – 550
- 551 – 600
- 601 – 650
- 651 – 700
- 701-750
- 751-800
- 801-850
- 851-900

73 - Do you live in the Nottingham area and would you like to participate in the basic cooking skills online sessions?

- Yes
- No
